# Nuclear Magnetic Resonance- and Electron Paramagnetic Resonance Spectroscopic Characterization of S_4_N_4_ and (SN)_*x*_ Dissolved in [EMIm][OAc]

**DOI:** 10.1021/acs.jpcb.5c00294

**Published:** 2025-04-10

**Authors:** Julian Radicke, Vanessa Jerschabek, Haleh Hashemi Haeri, Muhammad Abu Bakar, Dariush Hinderberger, Jörg Kressler, Karsten Busse

**Affiliations:** Department of Chemistry, Martin Luther University Halle-Wittenberg, von-Danckelmann-Platz 4, D-06120 Halle (Saale), Germany

## Abstract

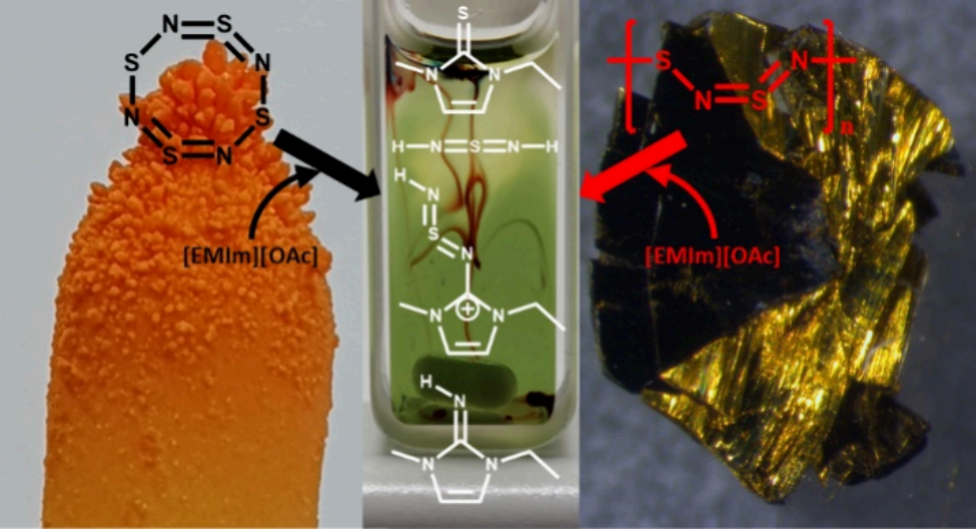

This work focuses on the reaction mechanism of poly(sulfur nitride) ((SN)_*x*_, (S^15^N)_*x*_), with the ionic liquid (IL) 1-ethyl-3-methylimidazolium acetate [EMIm][OAc]. We compare this with the reaction of the IL with S_4_N_4_ or its ^15^N-labeled form S_4_^15^N_4_, a precursor for the synthesis of (SN)_*x*_ and (S^15^N)_*x*_. After purification of the S_4_N_4_–IL- and S_4_^15^N_4_–IL systems via column chromatography, we characterized the reaction products with ^1^H, ^13^C, and ^15^N nuclear magnetic resonance spectroscopy and with electron spray ionization time-of-flight mass spectrometry. Furthermore, time-resolved electron paramagnetic resonance spectroscopy and time-resolved ultraviolet–visible spectroscopy were carried out. Thus, radical intermediates were detected, which were consumed with reaction time. Finally, we postulate a reaction mechanism for the S_4_N_4_–IL- and S_4_^15^N_4_–IL systems and compare this with the respective data for the (SN)_*x*_ −IL- and (S^15^N)_*x*_–IL-systems.

## 1 Introduction

Ionic liquids (ILs) and poly(sulfur nitride) (SN)_*x*_ are very interesting compounds with a plethora of applications. On the one hand, ILs act as solvents for a variety of polymers that cannot be dissolved in commercial solvents.^1−5^ On the other hand, (SN)_*x*_ is a simple constructed polymer, built of alternating sulfur and nitrogen atoms, and shows metallic conductivity.^6^ However, (SN)_*x*_ does not dissolve in conventional solvents, which complicates further examinations and modifications. In our first studies with 1-ethyl-3-methylimidazolium acetate [EMIm][OAc] as an IL, we observed reactive dissolution of (SN)_*x*_ together with color changes of the solution.

To clarify the interaction between both components, we performed prestudies^7^ on the kinetics of the reactive dissolution process of elemental sulfur (*cyclo*-S_8_) in [EMIm][OAc] which generated the corresponding 1-ethyl-3-methylimidazolium thione (EMImS). Taking into account the formation of reactive *N*-heterocyclic carbenes (NHCs), which are formed by imidazolium-based ILs,^8−15^ we were able to show that a nucleophilic attack from the carbene disrupts the sulfur ring system and degrades the S_8_–system stepwise.^16^ With the help of nuclear magnetic resonance (NMR), ultraviolet–visible (UV/vis), and continuous wave electron paramagnetic resonance (cw-EPR) spectroscopic data, it was also possible to detect intermediates that are formed and further converted during the reaction. In particular, the persistent trisulfide radical anion [S_3_]**^·–^** was observed which further reacts with [EMIm][OAc].^17−20^

The reaction of (SN)_*x*_^6,21−24^ in the same IL has several similarities with the system discussed above, but also significant differences. The NMR data showed a variety of reaction products, which were significantly more than those of S_8_ in [EMIm][OAc]. On the one hand, it could be determined that EMImS is also formed during the reaction and, on the other hand, further signals were found in the NMR spectra, which indicated imine-based compounds on the imidazolium ring.^25^ Another interesting difference in the sulfur conversion was indicated by EPR spectra. The examination via EPR spectroscopy requires a spin trap system using 5,5-dimethyl-1-pyrroline-*N*-oxide (DMPO) for obtaining significant signals, while the sulfur studies showed stable radicals without a spin trap. From the resulting data, we were able to conclude that although radicals are formed in the (SN)_*x*_–IL system, they are short-lived and not detectable without DMPO.

Here, the reaction of [EMIm][OAc] with tetrasulfur tetranitride (S_4_N_4_, S_4_^15^N_4_) will be investigated in detail, at first using the same spectroscopic methods as in our previous study.^7^ S_4_N_4_ is interesting because it is an important precursor for the polymerization of (SN)_*x*_ in which S_4_N_4_ reacts with silver wool to give S_2_N_2_ prior to the topo-chemical solid-state polymerization.^24,26−30^ The S_4_N_4_ forms a cage structure with two cross-ring S–S interactions, which provides suitable values for subsequent postulation of the reaction mechanism for the polymer. On the other hand, we could not investigate S_2_N_2_ directly because we did not isolate the monomer during the synthesis of (SN)_*x*_. Thus, we decided to examine the monomer precursors S_4_N_4_.^6,31^

Electrospray ionization time-of-flight mass (ESI-ToF-MS) spectrometry measurements are used as a further characterization method in this work, which provides a more detailed view of the product distribution and allows us to make a more specific assertion about the resulting products. From all characterization data, a logical reaction mechanism for the S_4_N_4_–IL system will be suggested. Finally, this allows the postulation of a reaction mechanism of the polymer (SN)_*x*_–IL system with [EMIm][OAc].

## 2 Experimental Section

### 2.1 Materials

1-Ethyl-3-methyl imidazolium acetate ([EMIm][OAc], abcr, 98.0%), dimethyl sulfoxide-d6 (DMSO-d6, abcr, 99.8 atom % d), toluene (Carl Roth, ≥99.5%), ethanol (Carl Roth, ROTIPURA ≥ 99.8%), activated carbon (Merck), silica gel 60 (Carl Roth, 0.03–0.2 mm), 5,5-dimethyl-1-pyrrolin-*N*-oxide (DMPO), methanol (Carl Roth, ROTISOLV HPLC Ultra Gradient grade), S_4_N_4,_^21,32^ S_4_^15^N_4,_^24,32^ (SN)_*x*_,^21,33,34^ and (S^15^N)_*x*_^24,33,32,34^ (synthesis details of S_4_N_4_ and (SN)_*x*_ described in Supporting Information Chapter 1).

### 2.2 Characterization Methods

#### 2.2.1 Nuclear Magnetic Resonance Spectroscopy

For the ^1^H, ^13^C, and ^15^N NMR spectroscopic studies of S_4_N_4_ and (SN)_*x*_ as well as the S_4_^15^N_4_ and (S^15^N)_*x*_ in [EMIm][OAc] and their isolated reaction products, an Agilent Technologies 500 MHz DD2 spectrometer was used at *T* = 27 °C. The 2D NMR experiments were performed with the same instrument.

The spectroscopic studies of the samples in the IL were performed with coaxial inserts for NMR tubes containing the calibration solvent DMSO-*d*_6_. NMR samples were prepared under a nitrogen atmosphere. The ^15^N NMR spectra were calibrated to the ammonia standard. MestReNova software (version 9.0) was used to evaluate all spectra. For NMR sample preparation, 25 mg of the isolated products after the reaction of S_4_N_4_/S_4_^15^N_4_ and (SN)_*x*_/(S^15^N)_*x*_, respectively, with [EMIm][OAc] were dissolved in 0.65 mL DMSO-*d*_6_.

#### 2.2.2 Electron Paramagnetic Resonance Spectroscopy

Room-temperature continuous wave electron paramagnetic resonance (CW-EPR) measurements at the X-Band frequency of 9.4 GHz were performed on a Magnettech MiniScope MS400 benchtop spectrometer (Magnettech, Berlin, Germany, now Bruker BioSpin). EPR spectra were recorded with a microwave power of 10 mW, 100 kHz modulation frequency, modulation amplitude of 0.2 mT, and 4096 data points. Each of the spectra is an accumulation of six scans, and each took 30 s. For simulations of EPR spectra, the easyspin software package (version 6.0.0-dev.51) was used.

All samples for EPR measurements were prepared under a nitrogen atmosphere. First, activated carbon (AC) (14.6 mg) and DMPO (4.2 mg; 0.037 mmol) were weighed in a glass vial. Then, [EMIm][OAc] (401.8 mg; 2.361 mmol) was added to the activated carbon with DMPO. The solution was stirred for a few minutes, and then the activated carbon was filtered off. In the next step, the filtrate was added to the S_4_N_4_ (2.9 mg; 0.016 mmol). The resulting mixture was taken up into a capillary, sealed with Critoseal, and measured. Following the same procedure, the samples for S_4_^15^N_4_ (2.6 mg; 0.014 mmol) in IL (428.2 mg; 2.516 mmol) with activated carbon (14.7 mg) and DMPO (5.5 mg; 0.049 mmol) and (S^15^N)_*x*_ (2.1 mg; 0.011 mmol) in IL (406.1 mg; 2.386 mmol) with activated carbon (18.3 mg) and DMPO (9.8 mg; 0.087 mmol) were prepared and measured.

#### 2.2.3 UV/vis Spectroscopy

The UV/vis absorption measurements were performed using a Hellma analytics quartz cuvette (*d* = 10 mm) on a PerkinElmer LAMBDA 365 UV/vis spectrophotometer. The temperature was controlled with a PerkinElmer Peltier System L365. First, a blank spectrum of [EMIm][OAc] (100 μL) was recorded with DMSO (2.0 mL) and set as a baseline. S_4_N_4_ (1 mg) was added to the solution and a spectrum was recorded between 300 and 700 nm at *T* = 20 °C. At the beginning, the spectra were recorded every 1 min, and after 30 min every 10 min. The data analysis was performed using OriginPro 2019.

#### 2.2.4 Electrospray Ionization Time-of-Flight Mass Spectrometry

ESI-ToF-MS spectrometry measurements were performed using a Focus Micro TOF spectrometer from Bruker Daltonics, which can operate in the positive as well as in the negative ion range. Both the ^15^N-labeled form (S_4_^15^N_4_) and the unlabeled form of S_4_N_4_ were dissolved in methanol (2.2 mg·mL^–1^). Then, 5 μL of the solution was diluted with 995 μL MeOH, filtered and measured. Subsequent analysis of the data was performed using Data Analysis software (version 4.0) and OriginPro 2019. In addition to the ^15^N enrichment, the natural isotope distribution was used for data evaluation.

### 2.3 Synthesis

#### 2.3.1 Reaction of S_4_N_4_ and S_4_^15^N_4_ with [EMIm][OAc]

Under a nitrogen atmosphere, S_4_N_4_ (31.7 mg; 0.172 mmol) was weighed in a glass vial and [EMIm][OAc] (751.0 mg; 4.412 mmol) was added. The glass vial was sealed with an aluminum cap and stirred for 48 h at *T* = 60 °C. The reaction solution was purified via column chromatography using a toluene/ethanol mixture (9:1 vol %/vol %). Subsequently, the solvent was removed via vacuum distillation. The mixture of products could be isolated as a reddish/brownish oily liquid. The same method was used to obtain the products of S_4_^15^N_4_ (27.0 mg; 0.143 mmol) in [EMIm][OAc] (706.9 mg; 4.153 mmol). ^1^H NMR (500 MHz, DMSO-*d*_6_, 27 °C): δ = 7.14 (d, ^3^*J* = 2.27 Hz 1H, HS–4), 7.11 (d, ^3^*J* = 2.44 Hz, 1H, HS–5), 6.48 (d, ^3^*J* = 2.44 Hz, 1H, HN–4), 6.43 (d, 1H,^3^*J* = 2.62 Hz, HN–5), 3.95 (q, ^3^*J* = 6.57 Hz, 2H, HS–7), 3.50 (q, ^3^*J* = 7.10 Hz, 2H, HN–7), 3.45 (s, 3H, HS–6), 3.09 (s, 3H, HN–6), 1.21 (t, ^3^*J* = 7.27 Hz, 3H, HS–8), 1.13 (t, ^3^*J* = 7.10 Hz, 3H, HN–8) ppm; ^13^C NMR (126 MHz, DMSO-*d*_6_, 27 °C): δ = 160.9 (C–2), 118.3 (C–5), 116.4 (C–4), 41.9 (C–7), 34.2 (C–6), 14.0 (C–8) ppm; ^15^N NMR (51 MHz, DMSO-*d*_6_, 27 °C): δ = 266.5, 265.8, 263.1, 262.5, 233.4, 233.2, 169.9, 169.0, 126.3, 110.0, 108.1, 95.7, 80.6, 58.0, 22.8, 20.2, 16.8, 15.4 ppm.

## 3 Results

### 3.1 Characterization of S_4_N_4_ and S_4_^15^N_4_ in [EMIm][OAc]

#### 3.1.1 NMR Spectroscopic Characterization

**Scheme 1:**
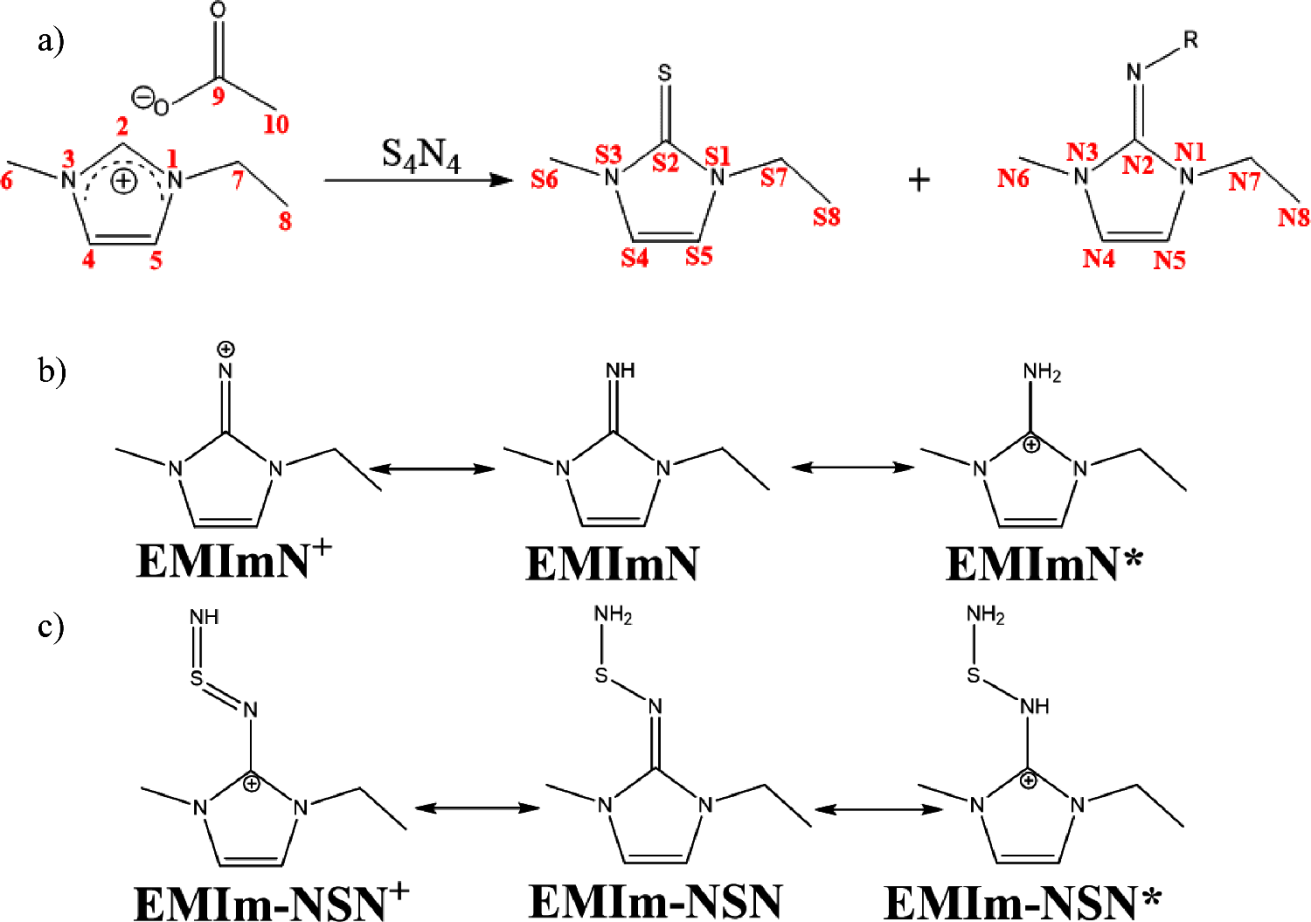
(a) The Reaction of [EMIm][OAc] with S_4_N_4_ Generates EMImS and an Imine Structure with Residue R (R = −H; −SH; =S=NH). (b) Protonated Variation of Products with EMImN and (c) Protonated Variations of EMIm-NSN

When S_4_N_4_ crystals are added to [EMIm][OAc] and stirred for 24 h at *T* = 60 °C, the subsequent NMR measurements reveal the same chemical shifts in ^1^H- and ^13^C NMR spectra, respectively, as observed in the previous work for the system (SN)_*x*_ in [EMIm][OAc].^7^ The ^1^H- and ^13^C NMR spectra (as well as the 2D NMR spectra) of the purified products of the S_4_N_4_–IL-reaction system showed the characteristic signals of 1-ethyl-3-methylimidazolium thione (EMImS).^7,16,35,36^ Also signals of a possible 1-ethyl-3-methylimidazolium imine compound^25,37^ as well as an unknown compound, probably also an imine compound, were identified (Supporting Information Figures S1 and S2 also Table S1). Scheme 1a shows educts and possible reaction products (EMImS and an imine structure with residue R in which R = −H, −SH, or =S=NH). Due to the large excess of [EMIm][OAc], we expect a proton-rich solution leading to several protonated states. In Schemes 1b and c, different protonation states of expected products are depicted.^38,39^

**Figure 1:**
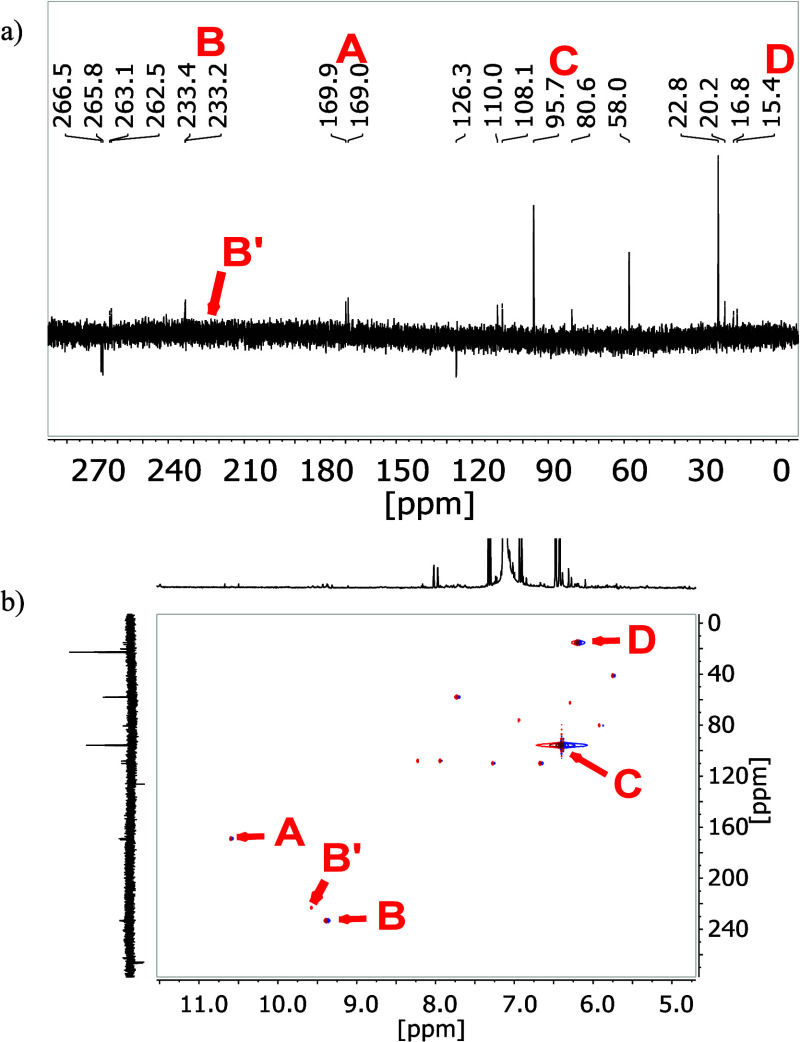
(a) Proton decoupled ^15^N NMR spectrum of the purified product of the reaction of ^15^N labeled S_4_^15^N_4_ with [EMIm][OAc] and (b) the corresponding ^1^H–^15^N HSQC NMR spectrum.

To analyze the imine structures further, we repeated the S_4_N_4_–IL reaction with ^15^N-labeled S_4_^15^N_4_. After purification, ^1^H and ^13^C NMR spectra (Supporting Information Figures S3 and S4) and also the ^15^N NMR spectrum (Figure 1a) were recorded. For the ^1^H and ^13^C NMR spectra, we were not able to see any difference between ^15^N labeled and unlabeled S_4_N_4_ in [EMIm][OAc].

**Scheme 2:**
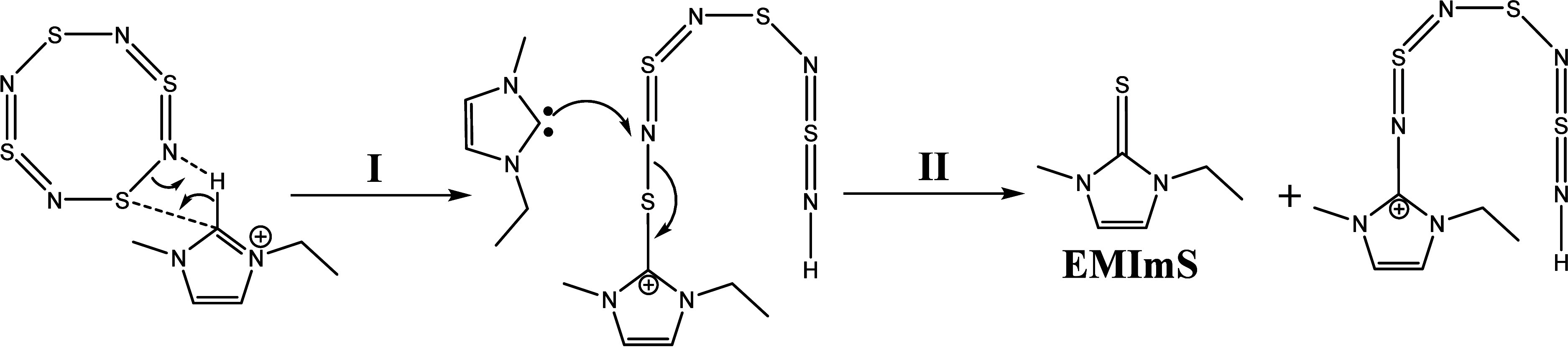
Postulated Initiation of the S_4_N_4_-Ring Opening by an Imidazolium Carbene

**Scheme 3:**
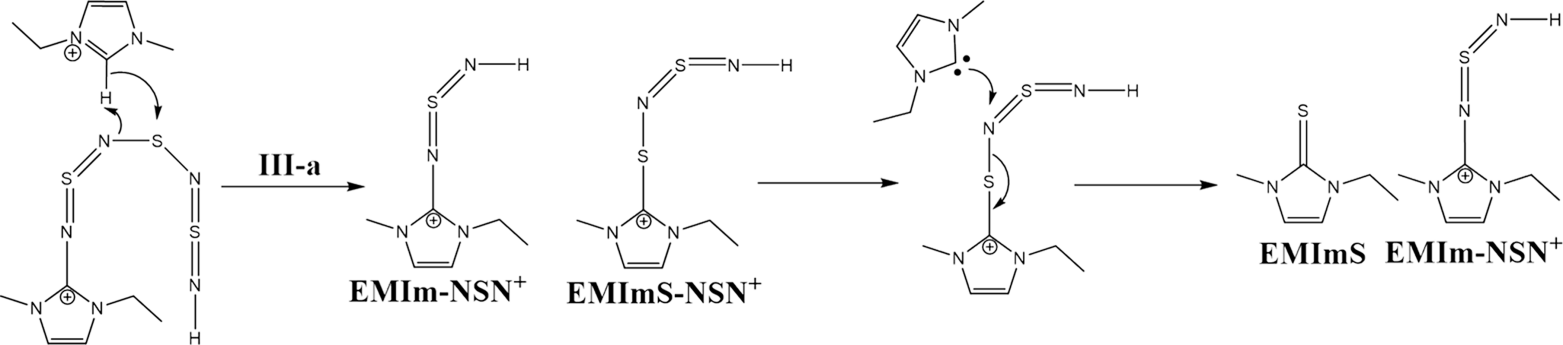
Postulated Reaction Mechanism Pathway **III-a**

**Scheme 4:**
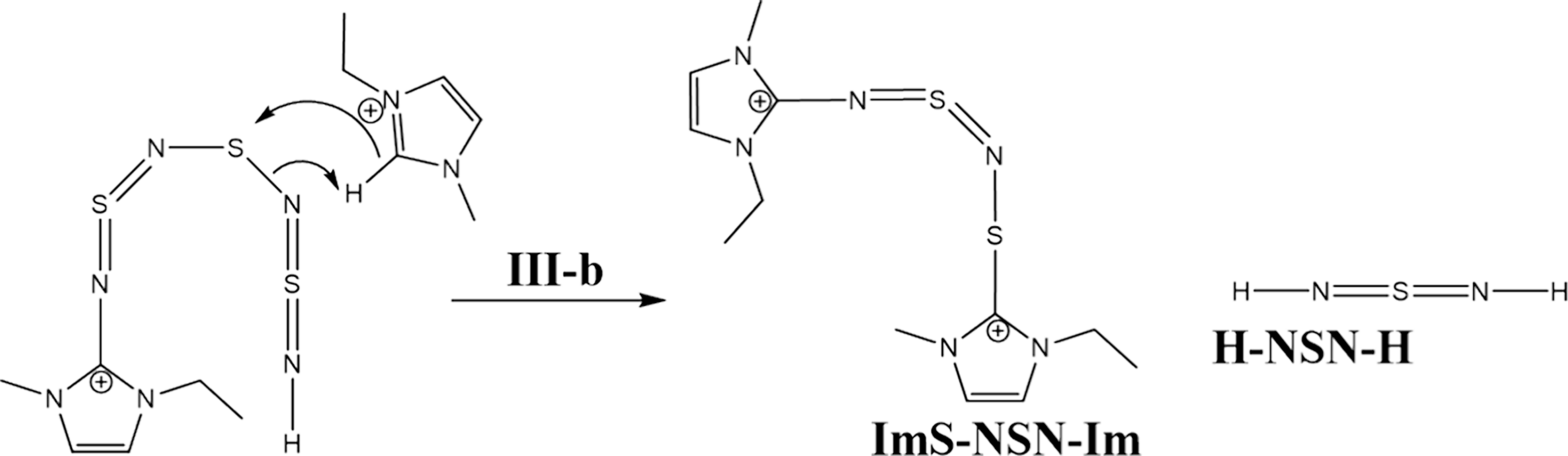
Postulated Reaction Mechanism of Step **III-b**

**Scheme 5:**
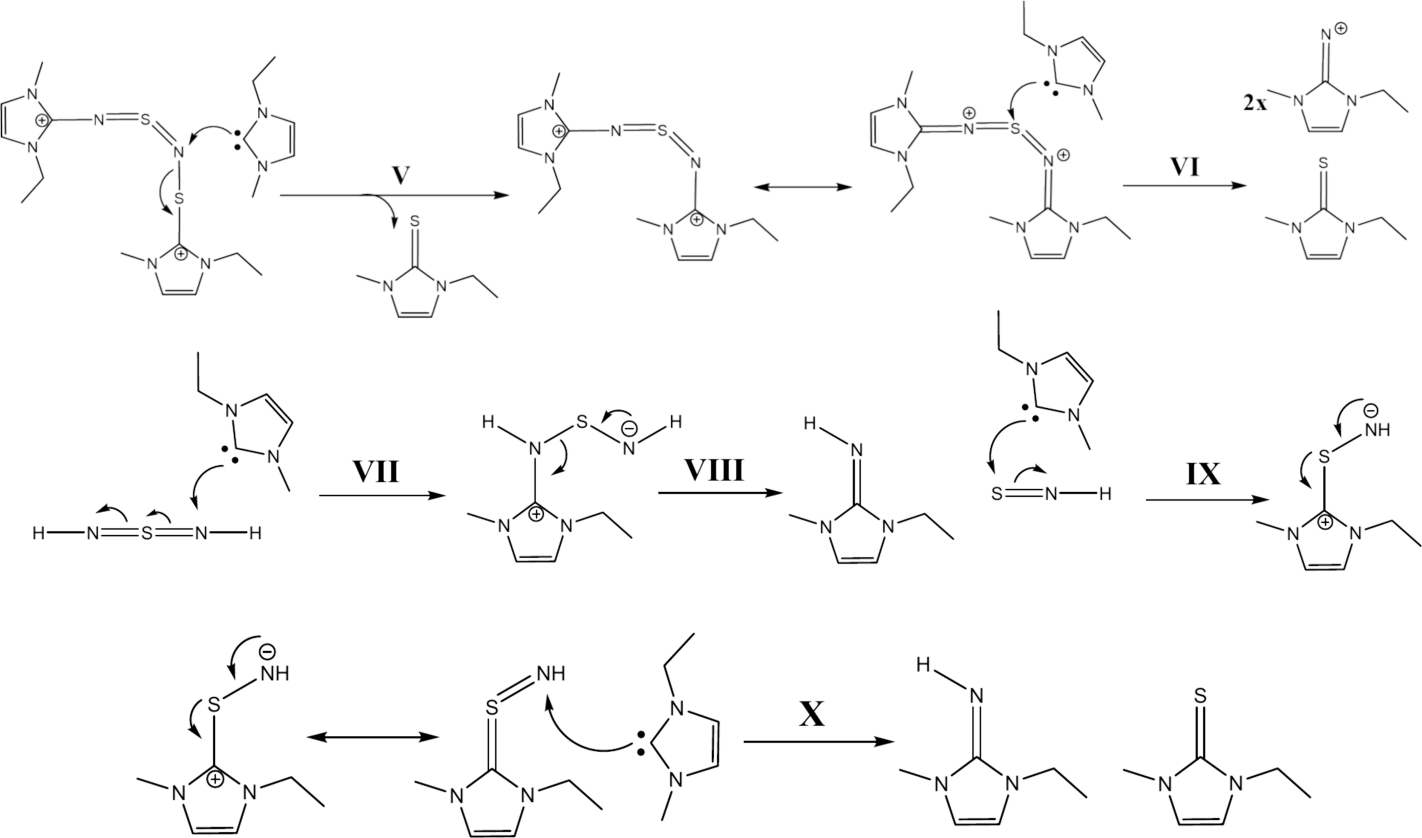
Reaction Steps **V** to **X** of the S_4_N_4_–IL Reaction

The proton decoupled ^15^N NMR spectrum showed many signals of imine or amine structures in the range between 0 and 280 ppm.^40−45^ The two strongest signals in the ^15^N NMR shift range between 0 and 120 ppm are indications that the nitrogen atom has a single bond with sulfur or carbon.^43,46^ The doublet signal in the ^15^N spectrum A at 169.9 and 169.0 ppm and the doublet signal B at around 233.2 and 233.4 ppm are clear indications for double bonds next to the nitrogen atom of the imidazolium ring. Furthermore, a splitting of the B signal in the ^15^N spectrum (^2^*J* (^15^N–^15^N) = 7.78 Hz) is an indication for an =NSN structure (EMIm-NSN^+^). The appearance of the small signal B′ is a hint that another molecule with an =N–H-group may exist. Furthermore, we can identify ^15^N signals in the spectrum indicating a broader product distribution than previously assumed. These are probably further protonated nitrogen atoms with a free electron pair or further intermediate products that are formed during the reaction and are not completely consumed (Supporting Information Scheme S1). This will be discussed in detail in the reaction Schemes 2–5.

Taking into account the ^1^H–^15^N 2D NMR spectrum (HSQC, Figure 1b), it is obvious that many ^15^N atoms in our products are protonated. The corresponding proton coupling constant for the signals A is ^1^*J* (^1^H–^15^N) = 86 Hz, which is typical for a coupling over one bond.^47^ Thus, the terminal NH groups of the molecules EMImN and EMIm-NSN^+^ are responsible for these signals. The signals at 95.7 ppm of C and 15.4 ppm D show clear proton couplings in the HSQC spectrum at 6.40 and 6.19 ppm with a respective coupling constant of ^1^*J* (^1^H–^15^N) = 72.0 Hz and ^1^*J* (^1^H–^15^N) = 89.0 Hz (Supporting Information Figure S5). These signals result from −NH_2_ groups of EMImN*, EMIm-NSN, and EMIm-NSN*.^40,47^ We cannot detect a N–H coupling signal of the secondary amine in EMIm-NSN*, so we assume that this molecule is not formed in a significant amount. Furthermore, we observe two double signals at 265.8/266.5 ppm and 262.5/263.1 ppm with a coupling constant of ^2^*J* (^15^N–^15^N) = 32.1 Hz and ^2^*J* (^15^N–^15^N) = 32.2 Hz (Figure 1b). These two double nitrogen signals show no proton coupling in HSQC NMR spectrum and we assume that these are tertiary nitrogens of EMImN^+^, EMIm-NSN^+^_,_ and EMIm-NSN (Scheme 1).^40,41,47−49^

#### 3.1.2 ESI-ToF-MS Characterization

**Figure 2:**
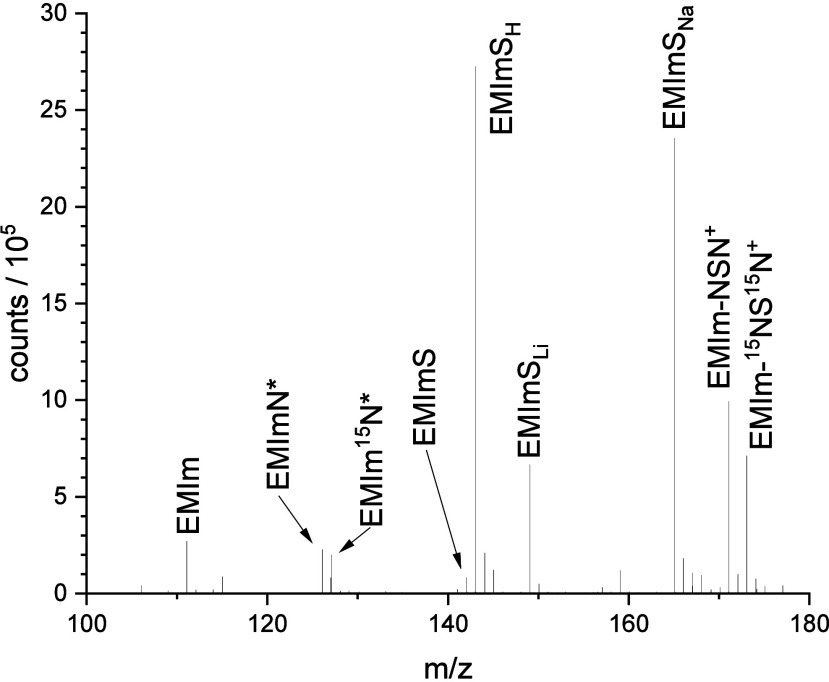
ESI-ToF-MS spectrum of the purified S_4_^15^N_4_–IL-system after reaction. The assignment is according to Table 1.

For further characterization, an ESI-ToF-MS measurement of the purified S_4_N_4_–IL reaction system was performed (Figure 2) to gain a better understanding for the reaction product distribution. It should be mentioned that during purification charged species were mostly adsorbed on the silica gel. Thus, the obtained molecule distribution does not necessarily represent completely all reaction products. Furthermore, due to the stepwise production of S_4_N_4_ with initially pure ^15^N-labeled NH_3_ followed by the finalization of the reaction with nonlabeled NH_3_, the reaction products should have an average isotope ratio of ^15^N to ^14^N of approximately 57–43 at % in the residue.

In the range of *m*/*z* between 100 and 180 of the ESI-ToF spectrum, five highly intense peaks and several smaller signals are detected. The three ionized thiones EMImS_X_ (with X = H, Li, Na) at *m*/*z* = 143, 149, and 165 (Table 1) give very intense signals. These ionized species are formed in the ESI-ToF chamber. In contrast, the signal at *m*/*z* = 171 and 173 belongs to imines with either two ^14^N or two ^15^N atoms (EMIm-NSN^+^), where no significant signals with Li or Na were found. These species are already ionized in the initial solvent (methanol). Other detected ions are 1-ethyl-3-methylimidazolium (EMIm) and protonated 1-ethyl-3-methyimidazolium imine (EMImN*), both without a significant amount of alkali metals.

**Table 1:**
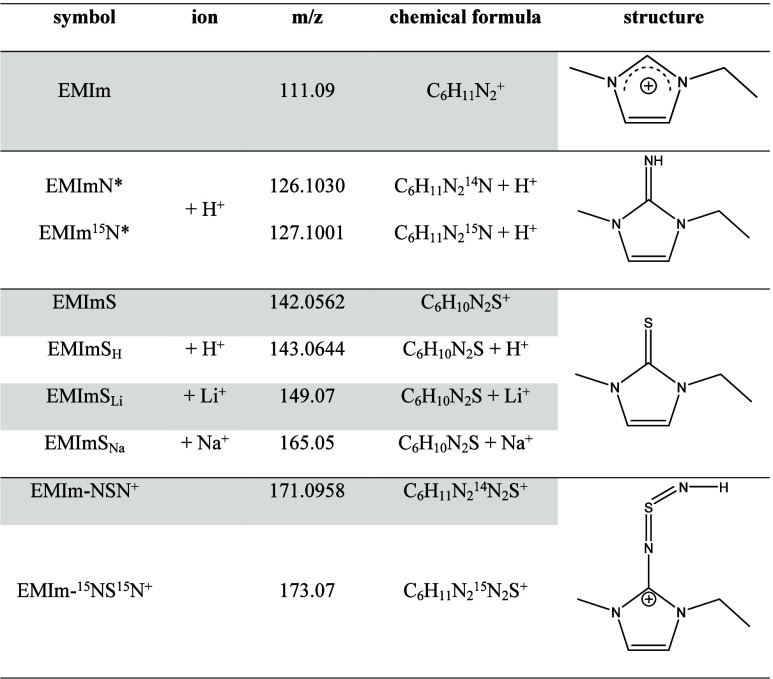
Possible Reaction Products of the S_4_N_4_–IL Reaction Solution with Mass-to-charge Ratio, Chemical Formula, and Chemical Structures

#### 3.1.3 UV/vis Characterization

In order to realize the UV/vis measurements and to minimize the influence of the absorption of [EMIm][OAc], DMSO was added to the S_4_N_4_–IL system under investigation. Initially, we performed a 30 min and a 4 h measurement for the S_4_N_4_–IL system in the range from 300 to 700 nm (complete spectra in Supporting Information Figure S7). The first UV/vis spectrum was recorded after adding S_4_N_4_ to the IL-DMSO solution and set as the zero-minute spectrum.

**Figure 3:**
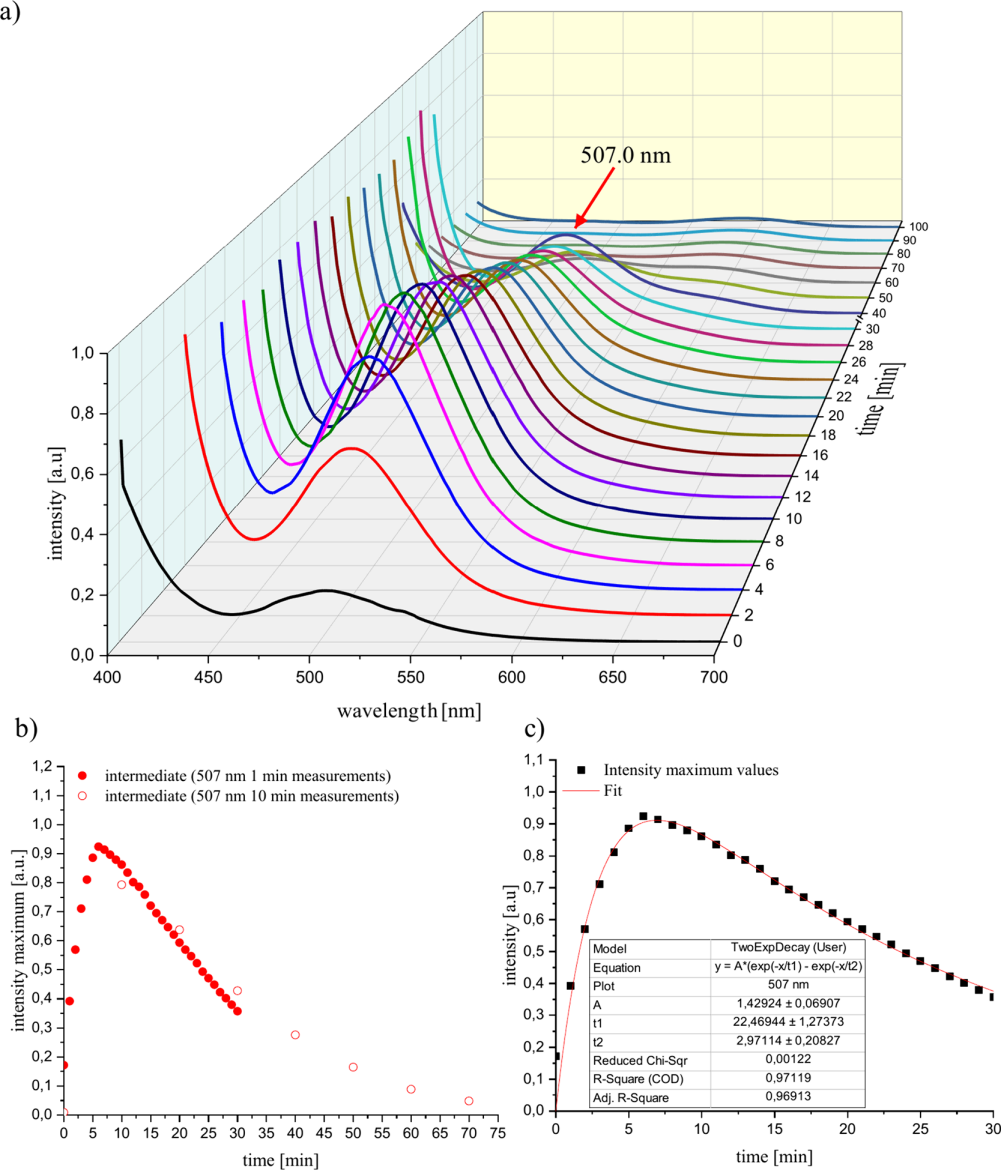
(a) Time-dependent UV/vis measurements of the reaction of S_4_N_4_ (1 mg) with [EMIm][OAc] (0.1 mL) in DMSO (2 mL). (b) Plot of the intensity maximum against time of the 30 min measurement compared with the 4 h measurement. (c) Kinetic fit of the intensity vs time plot.

An absorption band at 507 nm can be detected, which increases sharply in the first few minutes of the reaction. This was an indication of an intermediate produced by ring opening of S_4_N_4_ with the respective carbene. The decrease of this signal with time results from the further reaction of the intermediate (Figure 3b).

The reaction probably proceeds according to a scheme based on eq 1, in which the S_4_N_4_ (A) produces an intermediate compound B by ring opening, which further reacts to C in the next step. Assuming that the reaction is pseudo-first order and that the rate constants *k*_1_ and *k*_2_ are not equal, the kinetics can be described according to eq 2. Plotting the intensity maxima against time and the corresponding fit of the course of the absorption band according to eq 2 shows that the formation of the intermediate compound with a rate constant of *k*_1_ = 0.337 min^–1^ is much faster than the degradation of the intermediate compound with *k*_2_ = 0.0445 min^–1^ (Figure 3c).
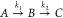
1
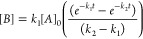
2

#### 3.1.4 EPR Characterization

The S_4_N_4_–IL system was examined using EPR spectroscopy to find out whether radical intermediates are formed during the reaction of S_4_N_4_ with [EMIm][OAc]. S_4_N_4_-based radical compounds are well-studied with a characteristic nine-line EPR spectrum, generated by electrochemical reduction of THF solutions of S_4_N_4_ below 0 °C.^50,51^

The radical ions are suggested to be very reactive and therefore one can consider them as short-lived species.^50−53^ Based on the measurements carried-out (Supporting Information Figure S8a,b), no evidence of radical intermediates was initially found, which implies two hypotheses, either no radicals are formed during the reaction or the generated radicals are short-lived and the subsequent reactions are correspondingly fast so that the radicals cannot be detected. To confirm or rule out the second hypothesis, we used the spin trap 5,5-dimethyl-1-pyrroline-*N*-oxide (DMPO) with the S_4_N_4_–IL system.

**Figure 4:**
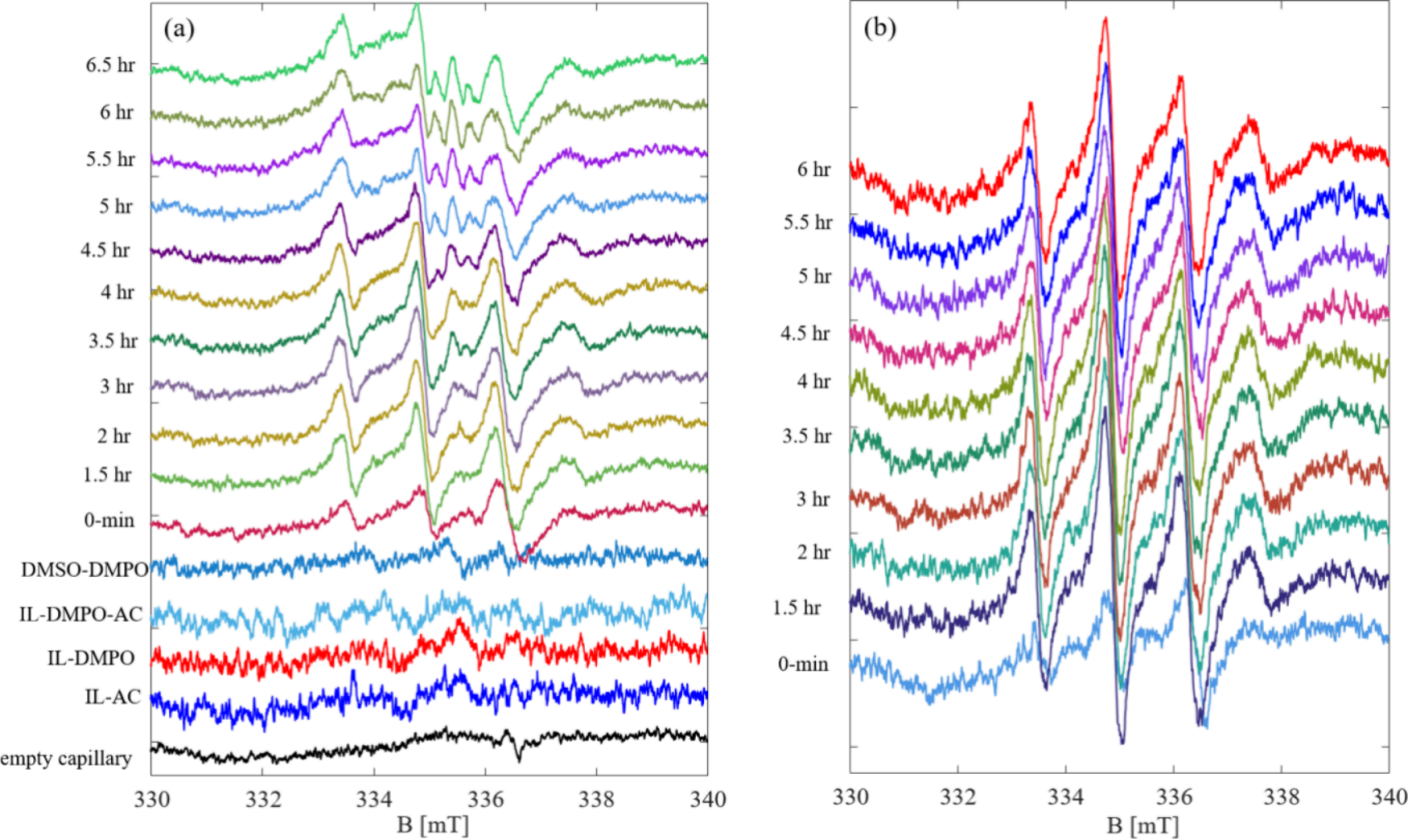
EPR spin trapping of a) S_4_N_4_ and b) S_4_^15^N_4_ taken over 6 h. Reference measurements of IL in the presence of activated carbon (AC), DMPO, and DMSO are also given.

We measured the EPR signals from the S_4_N_4_–IL-system over 6 h (Figure 4a). As can be seen, even at the start of the reaction (shown by 0 min), traces of the EPR signal are clearly observable. This implies a very fast radical generation. After 2 h, the signal reaches its maximum intensity and afterward, it starts decomposing to other radical species, reflected in a small splitting at around 335 mT. One might conclude that the radical intermediates are formed during the reaction of S_4_N_4_ with [EMIm][OAc] within 2 h and then they are consumed or decompose.

**Figure 5:**
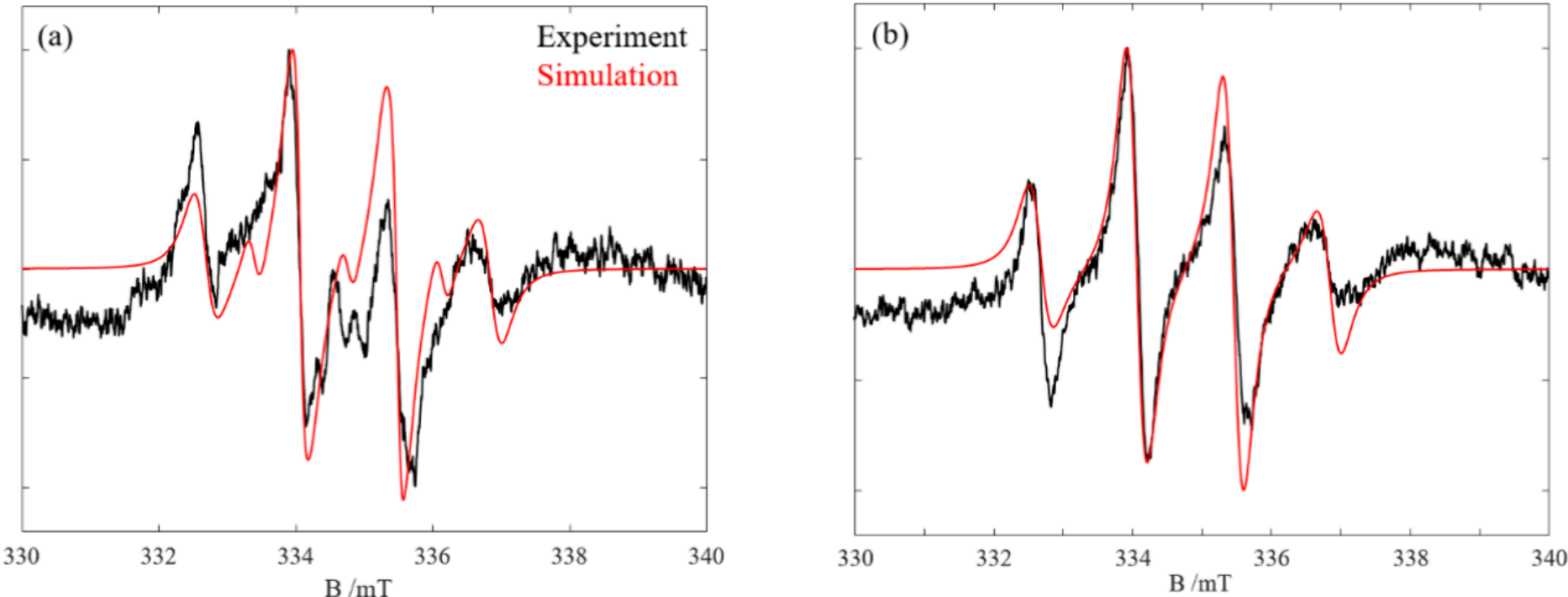
(a) EPR spin trapping of S_4_N_4_ and (b) S_4_^15^N_4_ in [EMIm][OAc] (in black) and corresponding simulations (in red).

For spectral simulations, we chose the spectrum recorded after 4.5 h, to be able to see different radicals formed (Figure 5). The experimental spectrum could be well simulated with *g*_iso_ = 2.012 and a three-component system, two fast tumbling components, A(^1^H) = 40.0 MHz (1.42 mT; 20%) and A(^14^N) = 38.6 MHz (1.38 mT; 10%) and a slow tumbling component with the same hyperfine coupling. This latter component also showed an exchange coupling of ∼8 MHz (0.28 mT). These findings are indicative of two points: high local concentration of radicals that reside in close proximity and the radical that binds to DMPO is a large molecular chain, which restricts its rotational movement and causes the suppressed high field line of the spectrum. In addition, it implies that a long-chain radical species is formed when the S_4_N_4_ ring is opened by the carbene, which can then attack the DMPO and provide an EPR signal. Moreover, we cannot rule out the formation of nitrogen-based radical species, which might also be formed during the reaction, and deliver an EPR signal via DMPO. The results are very similar to those that we found for spin trapping of (SN)_*x*_,^7^ albeit with slightly different contributions of the three different components. This shows that the monomeric SN units of (SN)_*x*_ and S_4_N_4_ follow the same type of reaction mechanism.

The experiment was repeated for the isotope labeled S_4_^15^N_4_ (Figure 4b). Spectral simulations for the recorded spectrum after 4 h of reaction, revealed no big difference from unlabeled S_4_N_4_. The broader spectral lines could be related to the nonresolved splittings of ^15^N. Considering both spectra, it can therefore be assumed that the reaction process is the same and similar intermediate compounds are formed. Simulated EPR spectra of spin trapped S_4_N_4_ and S_4_^15^N_4_ by DMPO are given in Figure 5. The S_4_N_4_ spectrum is simulated with a narrower line width to show the ^1^H splittings underneath the broad line width.

#### 3.1.5 Discussion

There are several investigations on reactions of different nucleophiles with S_4_N_4_ in the literature: Nucleophiles like triphenylphosphines PPh_3_ react with S_4_N_4_ and form a thione Ph_3_P = S and an imine Ph_3_P = N–S_3_N_3_.^54^ Chivers et al. described in several publications the mechanism of this reaction in which they postulated a ring-opening-mechanism which forms at first Ph_3_P = NS_3_N_3_. In the next step, this intermediate loses an NSN fragment and generates an open-chain intermediate Ph_3_P = N–SNS, which subsequently reacts with sulfur to give Ph_3_P = NSN = S=S.^30,55−58^ Giffin also examined the reactivity of carbenes with S_4_N_4_ and characterized the resulting products with X-ray diffraction measurements.^59^ Compared to Chivers et al., Giffin postulated for the reaction of imidazolidine-2-ylidene and imidazole-2-ylidene with S_4_N_4_ a similar mechanism pathway with a comparable product contribution. At first, the reaction generated a [carbene = NS_3_N_3_] product, which transformed to a [carbene = NSN = S=S] intermediate as well as postulated by Chivers et al. All publications that investigated the reaction of nucleophiles with S_4_N_4_ suggest a mechanism with the loss of an NSN fragment.

Our investigations include a similar system to that used by Chivers et al. and Giffin. We examine the reaction of imidazolium carbenes with S_4_N_4_ and postulate the corresponding mechanism. We also include a ring-opening scenario, which results in similar products (thione- and imine structures). However, the crucial difference between our investigations and previously published data is that we have a very high surplus of protons that exist in the IL [EMIm][OAc], which can protonate the nitrogen atoms of S_4_N_4_. We suggest that the reaction is initiated by the imidazolium-carbene. The concentration of carbene in pure [EMIm][OAc] is in the ppm range^60^ but high enough that it can react with sulfur–nitrogen compounds and generate the observed reaction products.^9,61,62^

NMR spectroscopic data showed that the reaction of S_4_N_4_ with [EMIm][OAc] produces a variety of reaction products. One of these was EMImS which we were also able to confirm by ESI-ToF-MS spectrometry. Other compounds were imidazolium imines with different residues on the nitrogen atom. On the one hand, an imine compound with a proton on the nitrogen was formed and on the other hand, the reaction generates imine structures with sulfur-amine and sulfur-imine residues (with a positive charge on carbon at position 2 in the imidazolium ring system). A comparison with literature data for the individual products showed that the signals occur at similar chemical shifts.^16,25,36,37,63^ All these structures were confirmed by ESI-ToF-MS. Accordingly, EMImS, the corresponding imine EMImN and other imine structures with a sulfur–nitrogen residue (EMIm-NSN) could be detected.

As mentioned earlier, Chivers et al. examined also the reaction of phosphorus(III) reagents with S_4_N_4_ and they postulated expulsion of an NSN fragment during the reaction.^55−58^ In comparison, our S_4_N_4_–IL system produces the corresponding thione, the imine, and other imidazolium based molecules with NSN fragments.^30,64^ UV/vis spectra showed an intermediate reaction product with a maximum at 507 nm and a decreasing concentration after 6 min. Thus, this species can be considered as an intermediate. Similar results were obtained by Chivers et al.^57^ with intermediate molecules having absorption bands between 478 and 485 nm (depending on the solvent). Thus, our intermediate could also be an N–S-fragment.

Based on our characterization data, we will postulate alternative reaction mechanisms for the conversion of S_4_N_4_ with the IL. All cases include an attack of the imidazolium-carbene on the two-coordinated sulfur atom. In step **I**, the S_4_N_4_ ring was opened by the attack of a proton (on position 2 from the imidazolium ring) with a nitrogen. The carbon (on position 2) forms a single bond with the sulfur atom. Subsequently, an intermediate is formed with a protonated nitrogen on one end of the chain and an imidazolium ring at sulfur at the other end (Scheme 2). This is possibly the intermediate observed in the UV/vis spectra. We do not assume a six-membered intermediate state as postulated by the mechanism discussed by Chivers et al.^30,55−57,59,65,66^ In step **II**, the resulting carbene attacks a nitrogen atom and forms EMImS and a compound with an imidazolium ring at the nitrogen. A similar compound with a long S_3_N_4_-chain was examined by Holt et al. and supports our assumption that such a molecule is generated during the reaction.^67^

Now, we have two possible reaction pathways (**III-a** and **III-b**) with this intermediate. Step **III-a** shows a protonation at the nitrogen atom of the EMIm-NSN fragment, which forms a cationic species EMIm-NSN^+^ (Scheme 3). The neighboring sulfur atom can attach an imidazolium ring and produce an EMImS-NSN^+^ fragment. After the next attack of a carbene at this fragment EMImS and an EMIm-NSN^+^ intermediate are formed. They can be detected by ESI-ToF-MS spectrometry.

Step **III-b** suggests the protonation of another nitrogen which produces the sulfur diimide H-NSN-H while the neighboring sulfur atom has an imidazolium ring residue. This molecule is a candidate for the B′ signal in the ^15^N NMR spectrum. This step generates a diimidazolium dicationic compound ImS-NSN-Im and an H-NSN-H fragment^68,69^ (Scheme 4).

In the next steps, the ImS-NSN-Im reacts with other carbenes. Then, two EMImS and two imidazolium imine compounds with positive charges are formed (step **V** to **VI**, Scheme 5). The sulfur diimide H-NSN-H can also react with carbenes (steps **VII** to **X**) and produce two imidazolium imines and one EMImS. However, the ImS-NSN-Im species from **III-b** could also follow another possible reaction mechanism (Supporting Information Chapter 5 step **S–IV** to **S–VII**). This results in the production of radical species which we might detect in the respective EPR measurements.

### 3.2 Characterization of (SN)_*x*_ and of (S^15^N)_*x*_ in [EMIm][OAc]

#### 3.2.1 ESI-ToF Spectrometry Characterization

**Figure 6:**
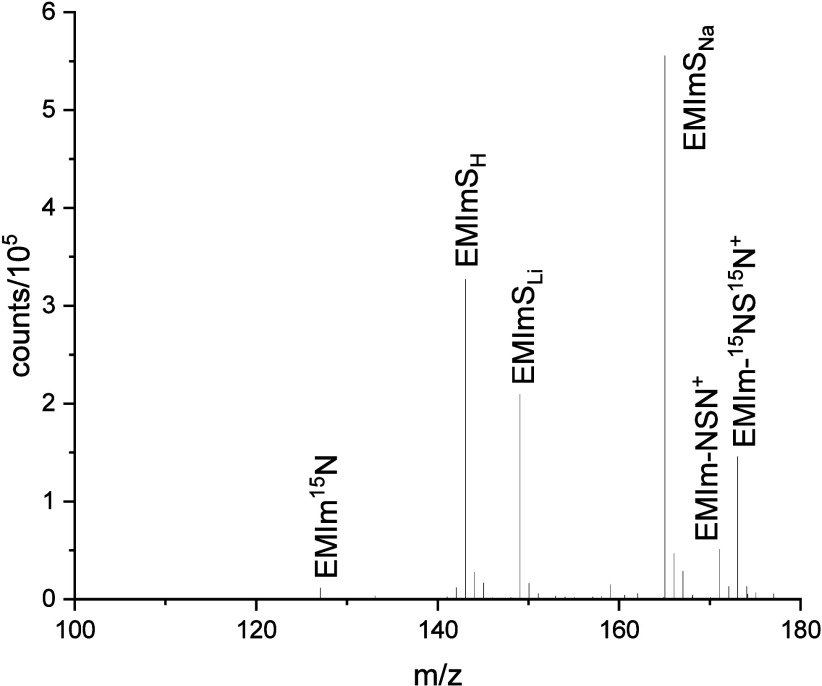
ESI-ToF-MS spectrum of the purified (S^15^N)_*x*_–IL-system after reaction. Assignment according to Table 1.

The ESI-ToF mass spectra of the purified (SN)_*x*_–IL- and (S^15^N)_*x*_–IL-systems after reaction are very similar except for the isotope distribution of nitrogen. As an example, Figure 6 shows a spectrum of the ^15^N-labeled system. A significant amount of the ionized thiones EMImS_X_ (with X = H, Li, Na) and imines EMIm-NSN^+^ (D, D′) with both nitrogen isotopes are observed. In contrast to the S_4_N_4_ results, almost no shorter imines EMImN (B,B′) were observed, but significantly more signals appear above *m*/*z* = 180, indicating larger molecular units of (S^15^N)_*x*_ attached to thiones or imines, or molecules with more than one EMIm attached (Supporting Information Figure S6).

#### 3.2.2 UV/vis Spectroscopy Characterization

**Figure 7:**
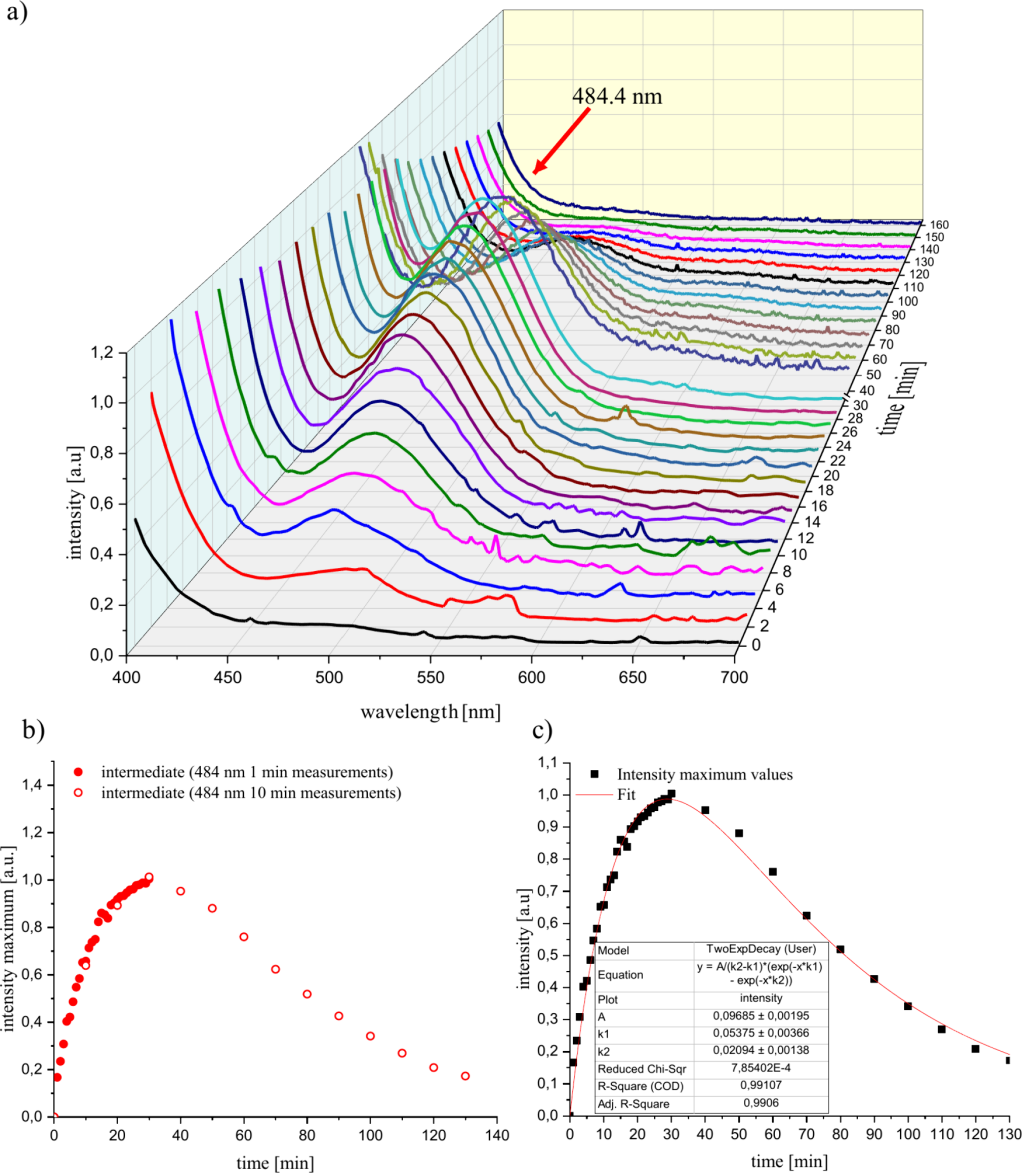
(a) UV/vis spectra dependent on time of (SN)_*x*_ (1 mg) in [EMIm][OAc] (100 μL) and DMSO (2.0 mL) and (b) time-dependent plot of the intensity maximum of the intermediate at 484 nm.

The characterization of the (SN)_*x*_–IL reaction via UV/vis spectroscopy showed an intermediate with an absorption band at 484 nm. We assume that the reaction kinetics follows the scheme given in eq 1. At the first step, the carbene attacks the polymer chain, breaks it, and forms an intermediate, which can be observed as an increase in intensity of the band at 484 nm (Figure 7a,b). This intermediate is probably also an imidazolium ring system with an N–S fragment similar to the intermediate from the S_4_N_4_–IL reaction. This was also described by Chivers et al.^56^

In the second step, the degradation of the intermediate starts and forms the resulting products. After plotting the intensity maxima against time and subsequent fitting of the graph according to eq 2 (Figure 7c), it shows that the formation of the intermediate with *k*_1_ = 0.054 min^–1^ is much faster than the degradation with *k*_2_ = 0.021 min^–1^.

#### 3.2.3 EPR Characterization

**Figure 8:**
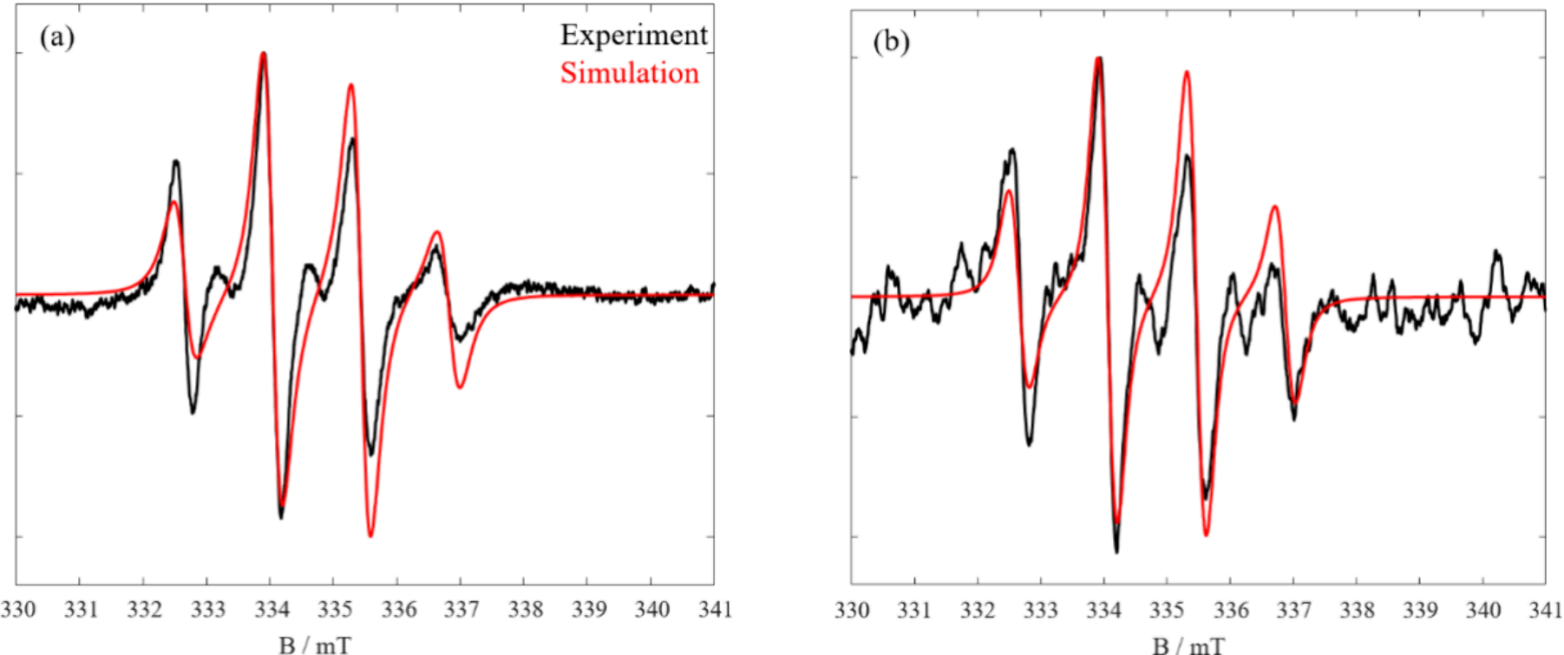
Experimental spectra (black) and corresponding simulations (red) of spin trapped (SN)_*x*_ and (S^15^N)_*x*_ in IL.

The EPR spectroscopic investigations of (SN)_*x*_ in [EMIm][OAc]^7^ show many similarities with the S_4_N_4_–IL system. Without a corresponding spin trap, no radical compounds could initially be detected (Supporting Information Figure S8c,d). Only when the investigations were carried out with DMPO, it was possible to detect potential radicals (Figure 8). The splitting pattern of the EPR spectrum is similar to that of the S_4_N_4_–IL system. Therefore, it is reasonable to conclude that the same radical intermediates are formed in the (SN)_*x*_–IL system. Also the EPR spectrum of (S^15^N)_*x*_ did not add any new information. In fact, both spectra can be simulated with one component, composed of one ^1^H and one ^14^N nuclei with hyperfine couplings of A(^1^H) = 38.6 MHz (1.38 mT) and A(^14^N) = 40 MHz (1.42 mT), respectively. It shows that for polymers the spin density is localized more on ^14^N rather than ^1^H, as was the case in the S*_4_*N_4_ system. Similar to the EPR observations for the S_4_N_4_ system, the exchange coupling of ∼8 MHz (0.28 mT) could be seen for (SN)_*x*_ and (S^15^N)_*x*_ polymers.

Again, the damped high-field peak of the spectrum is due to exchange coupling, which could be reasonably simulated for both spectra with about 3.5 MHz. These findings indicate that in both systems, with and without ^15^N-labeled nitrogen, long-chain radical intermediates are formed, which are consumed in the further course of the reaction to form the end products. The EPR spectra as a function of reaction time can be found in Supporting Information Figures S9–S10.

#### 3.2.4 Discussion

**Scheme 6:**
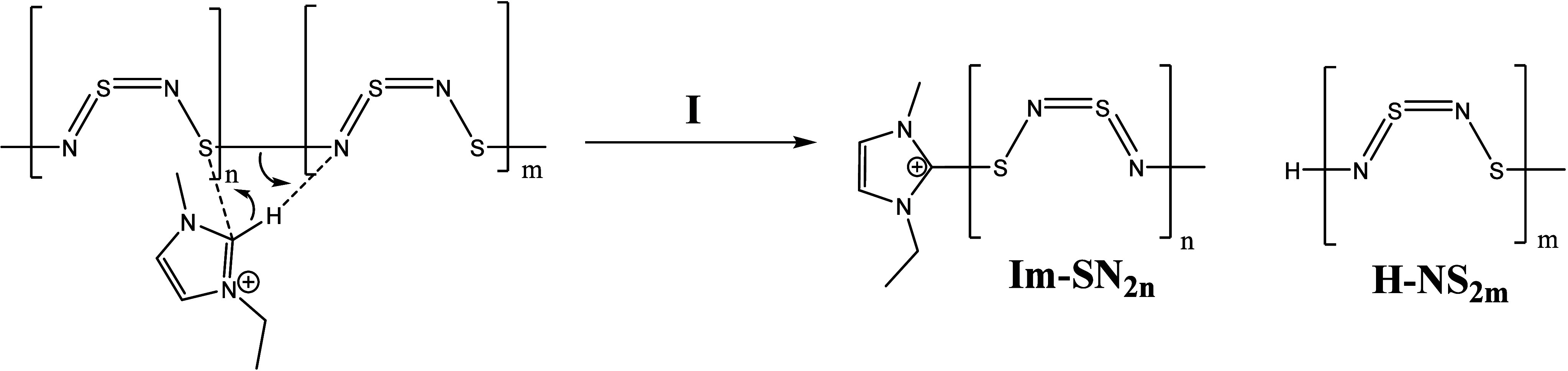
Initiation of the (SN)_2*x*_ Chain Scission with [EMIm][OAc]

**Scheme 7:**
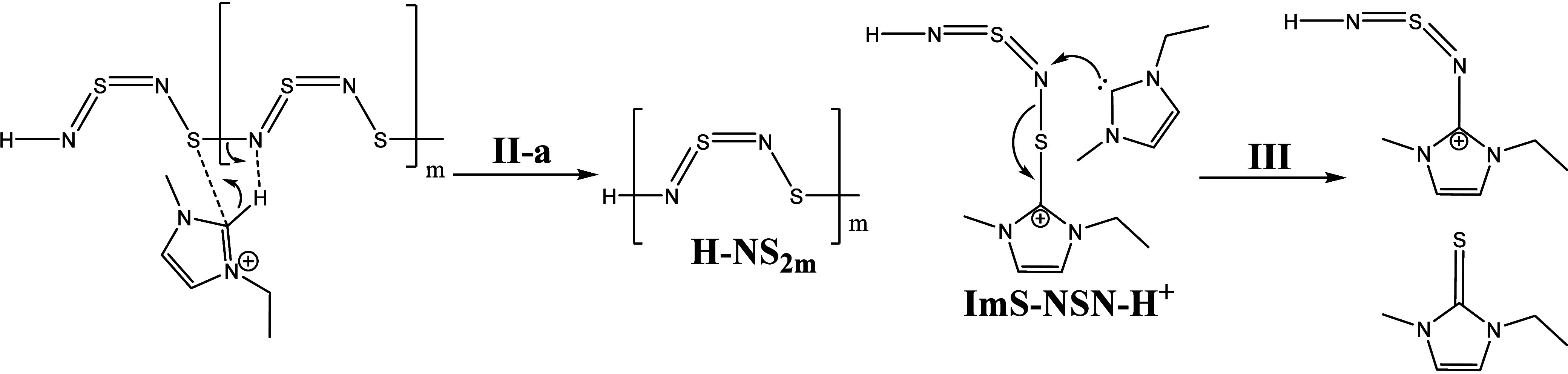
Steps **II-a** and **III** of the (SN)_2*x*_–IL System

**Scheme 8:**
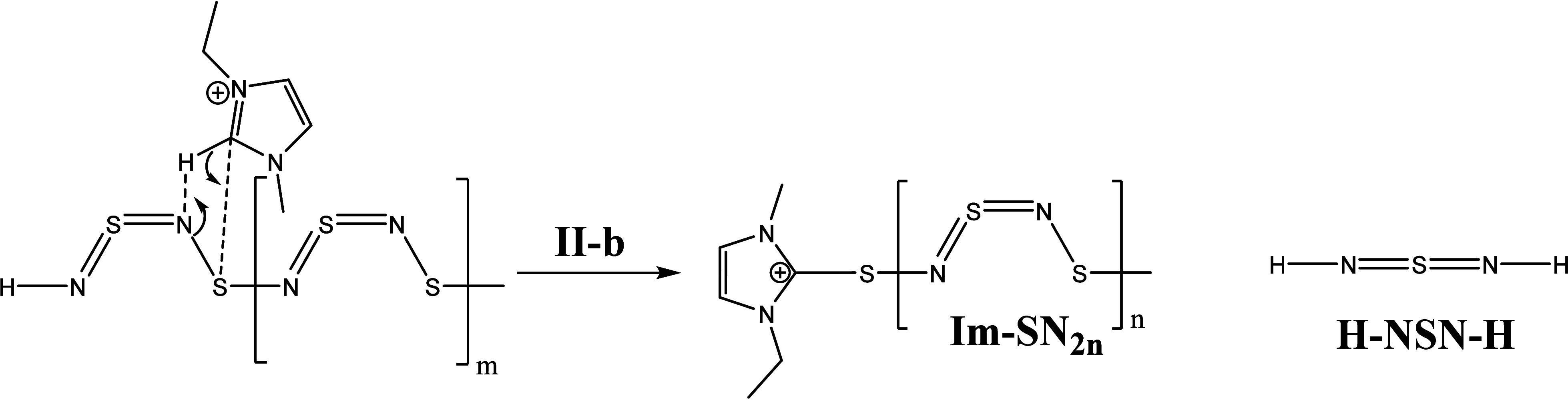
Reaction Steps **II-b** of the (SN)_2*x*_–IL System

**Scheme 9:**
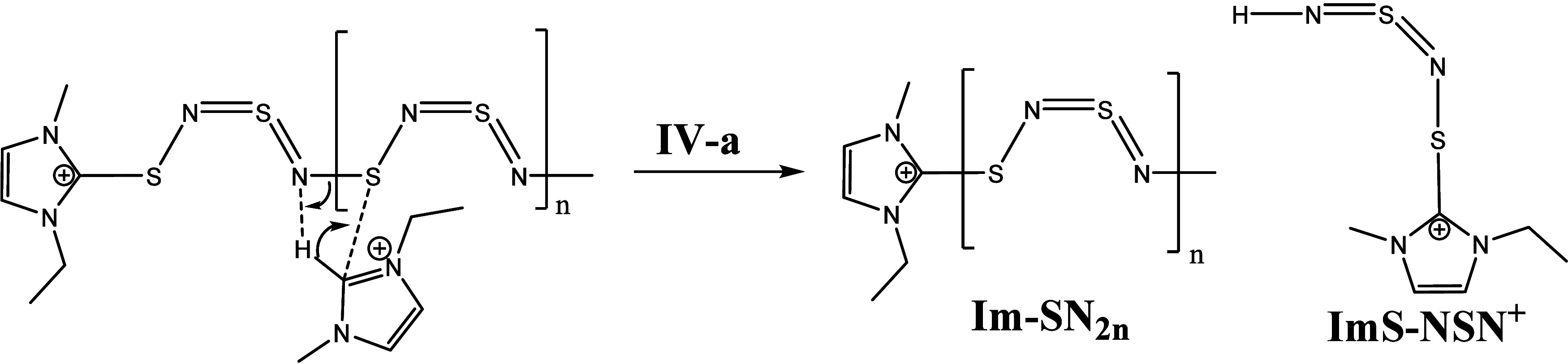
Step **IV-a** of the (SN)_2*x*_–IL Reaction

**Scheme 10:**
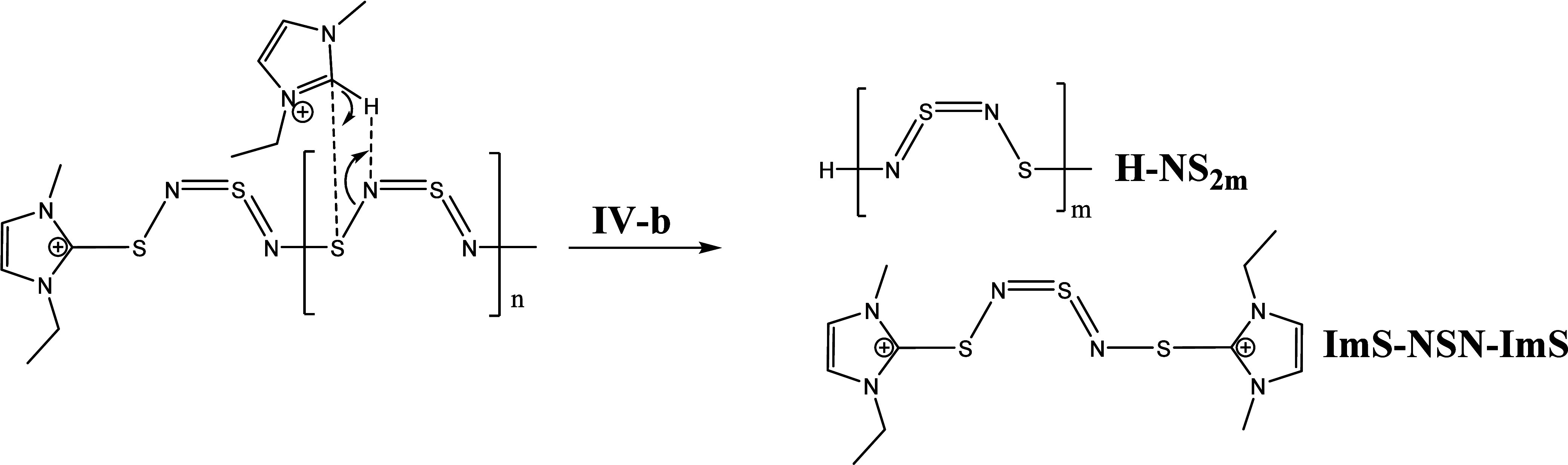
Step **IV-b** of the (SN)_2*x*_–IL System

**Scheme 11:**
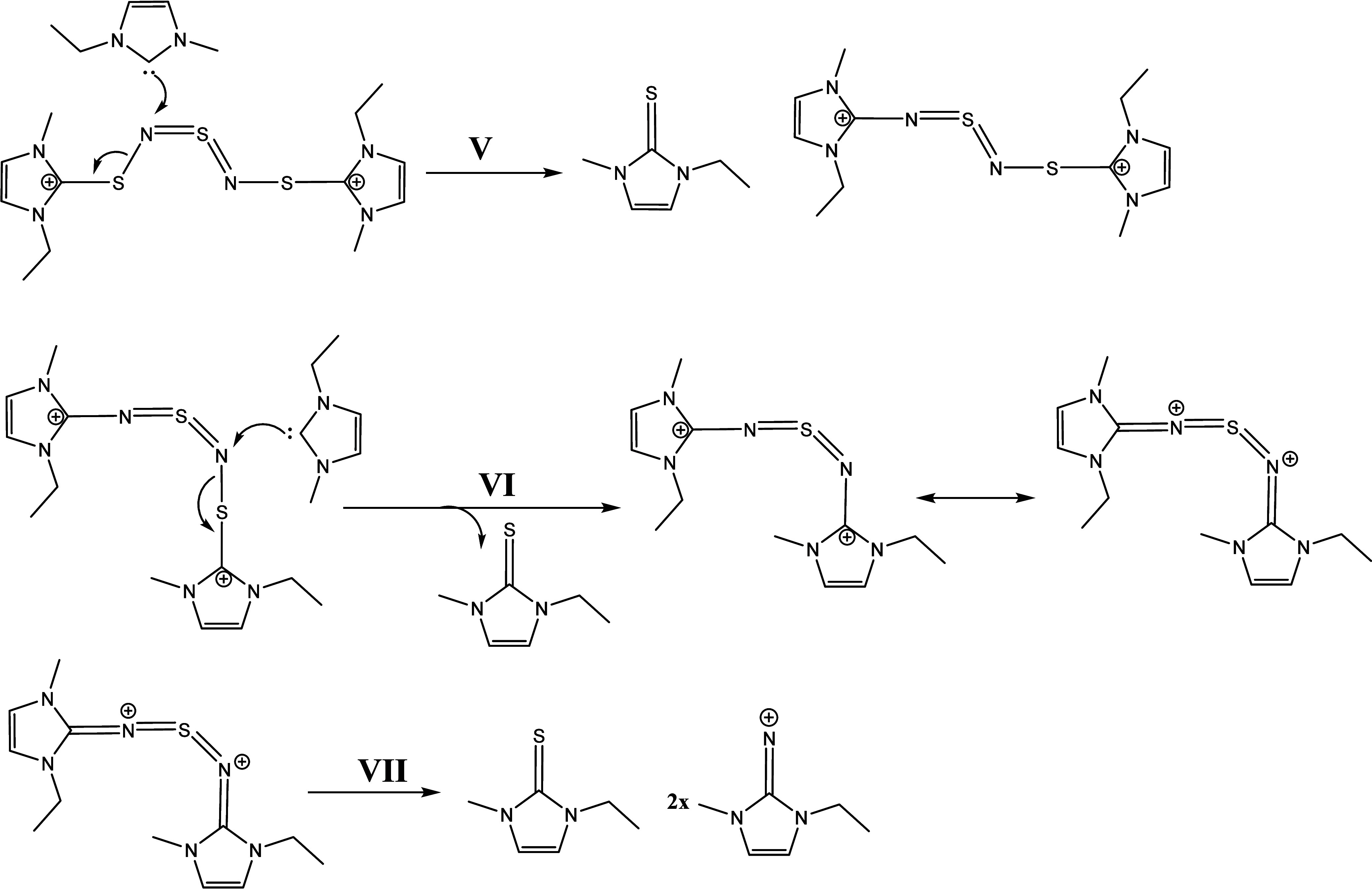
Step **V** to **VII** of the (SN)_2*x*_–IL Reaction

Based on the characterization methods (NMR, ESI-ToF, UV/vis, and EPR) and in comparison, with the reaction mechanism of the S_4_N_4_–IL system, we postulate a reaction mechanism of the (SN)_*x*_–IL system (Scheme 6–11). The reaction starts with the chain scission of the (SN)_*x*_ polymer (step **I**). This is caused by the attack of an imidazolium cation and generates two polymer fragments (Scheme 6). The first fragment with **n** numbers of SN-monomer units has an imidazolium cation molecule as end group (Im-(SN)_2*n*_) and the second fragment with **m** numbers of SN-monomer units has a proton at a nitrogen as end group (H-(NS)_2*m*_). Then, both polymer intermediates can be further degraded in different ways.

The H-(NS)_2*m*_ intermediate could react with an imidazolium cation by two possible steps **II-a** (Scheme 7) and **II-b** (Scheme 8). For the reaction **II-a**, the polymer chain breaks between a sulfur and a nitrogen atom and generates an H-(NS)_2*m*_ fragment and an ImS-NSN-H^+^ intermediate (as in the S_4_N_4_ reaction). In the next step **III**, ImS-NSN-H^+^ can react with the carbene and produce EMImS and EMIm-NSN^+^ which we detected in the ESI-ToF spectra.

Step **II-b** postulates a protonation at the second nitrogen (next to the H in terminal position) which results in the Im-(SN)_2*n*_ fragment and the sulfur diimide H-NSN-H,^68^ which can further degrade via step **VII** to step **X**. in Scheme 5.

In the following Schemes 9 and 10, we discuss the reaction steps of the Im-(SN)_2*n*_ intermediate. Also here, we have two possible mechanisms **IV-a** and **IV-b**. In step **IV-a**, we propose a mechanism in which the second nitrogen (next to the Im in terminal position) is protonated. The polymer chain is broken and splits into an Im-(SN)_2*n*_ fragment and an ImS-NSN^+^ compound. The Im-(SN)_2*n*_ fragment can then further react.

Reaction step **IV-b** suggests a protonation at the third nitrogen next to the terminal Im group and a substitution of an imidazolium cation at the third sulfur atom. The resulting compounds of this mechanism are an H-(NS)_2*m*_ polymer fragment and an ImS-NSN-ImS intermediate.

The ImS-NSN-ImS intermediate can further react with two carbenes (step **V** to **VII**, Scheme 11) and generates two EMImS compounds (observed in the ESI-ToF spectrum) and an Im-NSN-Im fragment. This fragment finally degrades with a carbene to EMImS and two imidazolium imine cations (detected by NMR and ESI-ToF). Another possible mechanism for the degradation of the ImS-NSN-Im which results in radical intermediates can be found in Supporting Information Chapter 6.

## 4 Conclusions

We started our examinations with the goal to find a solvent for (SN)_*x*_. As ILs are very good solvents for different polymers, we tested several ILs and observed a dissolution of (SN)_*x*_ in [EMIm][OAc].^7^ Unfortunately, it was a reactive process and the (SN)_*x*_ was consumed. To understand the reaction mechanism, we performed preliminary studies with S_8_ and S_4_N_4_ before the reaction with (SN)_*x*_. was studied in detail.

We were able to show that the reaction of S_4_N_4_ (S_4_^15^N_4_) in [EMIm][OAc] leads to several reaction products. Among others, the EMImS and various imine structures could be detected by NMR and ESI-ToF-MS spectroscopy. The UV/vis analysis showed that an intermediate is initially formed during the reaction, which is subsequently degraded. EPR measurements with DMPO as spin trap also showed that this intermediate is a radical compound formed by the ring opening of S_4_N_4_. According to the experimental data, we then postulated the possible reaction mechanisms (Schemes 2–5).

The (SN)_*x*_–IL ((S^15^N)_*x*_–IL) system showed the same final product distribution as the S_4_N_4_–IL system (according to NMR and ESI-ToF data), plus several higher mass fragments. Also, the EPR spectra showed similar intermediates when S_4_N_4_ or (SN)_*x*_ react with the IL. In both cases, we saw a damped high-field peak which suggests that we have a long-chain radical intermediate. Finally, we postulate possible reaction mechanisms (Scheme 6–11) which involve a protonation step with release of a carbene, a fragmentation of the polymer chain and possible reactions of the intermediates.

Obviously, [EMIm][OAc] is not a purely physical solvent for (SN)_*x*_ as the NHC reacts with the polymer. But if the NHC formation can be reduced by suitable anions or modification of the cation, then ILs might act as physical solvents.

## Supporting Information Available

In Chapter 1, we explain the synthesis of S_4_N_4_/S_4_^15^N_4_ and (SN)_*x*_/(S^15^N)_*x*_. Figures S1 and S2 shows the ^1^H, ^13^C, HSQC, and HMBC NMR spectra of purified products of the S_4_N_4_–IL reaction and Figures S3 and S4 show the NMR characterization of the S_4_^15^N_4_–IL system; Table S1 shows the chemical shifts of (SN)_*x*_ and S_4_N_4_ in [EMIm][OAc] and of the purified products; Figure S5 shows the ^1^H–^15^N-HSQC and HMBC NMR spectra of the products of the S_4_^15^N_4_ reaction with [EMIm][OAc]; Scheme S1 shows possible tautomeric forms of the EMIm-NSN compound and other products. Figure S6 shows the complete ESI-ToF-MS spectrum of the (S^15^N)_*x*_ reaction products; Figure S7 displays the complete UV/vis spectra of the S_4_N_4_–IL reaction. The reference EPR spectra of S_4_N_4_/S_4_^15^N_4_ and (SN)_*x*_/(S^15^N)_*x*_ in [EMIm][OAc] without DMPO are given in Figure S8 and the time-dependent EPR measurements of (SN)_*x*_/(S^15^N)_*x*_ are shown in Figures S9 and S10; Chapter 5 describes other possible reaction mechanisms with radical intermediates of the reaction of S_4_N_4_ and (SN)_*x*_, respectively, in the IL (PDF)

## Abbreviations

[A]_0_: start concentration of educt
[B]: concentration of intermediate
[EMIm][OAc]: 1-ethyl-3-methylimidazolium acetate
^14^N: nitrogen-14
^15^N: nitrogen-15
*d*: doublet
δ: chemical shift
DMPO: 5,5-dimethyl-1-pyrroline-N-oxide
DMSO-d6: deuterated dimethyl sulfoxide
EMImN: 1-ethyl-3-methylimidazolium imine
EMImS: 1-ethyl-3-methylimidazolium thione
EPR: electron paramagnetic resonance
ESI-ToF-MS: electron spray ionization time-of-flight mass spectrometry
g_iso_: lande factor
h: hour
HMBC: heteronuclear multiple bond correlation
HSQC: heteronuclear single quantum correlation
Hz: hertz
IL: ionic liquid
*J*: coupling constant
K: kelvin
k: reaction rate constant
Li: lithium
*m*/*z*: mass-to-charge ratio
mg: milligram
min: minute
mL: milliliter
Na: sodium
NHC: *N*-heterocyclic carbene
NIR: near-infrared
nm: nanometer
NMR: nuclear magnetic resonance
ppm: parts per million
*q*: quartet
*s*: singlet
S_2_N_2_: disulfur dinitride
S_4_N_4_: tetrasulfur tetranitride
(SN)_*x*_: poly(sulfur nitride)
T: temperature
*t*: triplet
t: time
UV/vis: ultraviolet–visible
wt %: weight percent
