# Supplementary material for: Nuclear Magnetic Resonance- and Electron Paramagnetic Resonance Spectroscopic Characterization of S4N4 and (SN)x Dissolved in [EMIm][OAc]
Source: J Phys Chem B. 2025 Apr 10;129(16):4063–77. doi: 10.1021/acs.jpcb.5c00294 (PMC12035862; doi:10.1021/acs.jpcb.5c00294)
Supplement: Supplementary file 1 — jp5c00294_si_001.pdf [file jp5c00294_si_001.pdf]

Supporting Information of

NMR- and EPR Spectroscopic Characterization of S_4N_4 and $(SN)_x$ Dissolved in [EMIm][OAc]

*Julian Radicke[a], Vanessa Jerschabek[b], Haleh Hashemi Haeri[c], Muhammad Abu Bakar[d], Dariush Hinderberger[e], Jörg Kressler[f] and Karsten Busse[g]**

[a]Department of Chemistry, Martin Luther University Halle-Wittenberg, von-Danckelmann-Platz 4, D-06120 Halle (Saale), Germany;

[b]Department of Chemistry, Martin Luther University Halle-Wittenberg, von-Danckelmann-Platz 4, D-06120 Halle (Saale), Germany;

[c]Department of Chemistry, Martin Luther University Halle-Wittenberg, von-Danckelmann-Platz 4, D-06120 Halle (Saale), Germany;

[d]Department of Chemistry, Martin Luther University Halle-Wittenberg, von-Danckelmann-Platz 4, D-06120 Halle (Saale), Germany;

[e]Department of Chemistry, Martin Luther University Halle-Wittenberg, von-Danckelmann-Platz 4, D-06120 Halle (Saale), Germany;

[f]Department of Chemistry, Martin Luther University Halle-Wittenberg, von-Danckelmann-Platz 4, D-06120 Halle (Saale), Germany;

[g]Department of Chemistry, Martin Luther University Halle-Wittenberg, von-Danckelmann-Platz 4, D-06120 Halle (Saale), Germany; Email: karsten.busse@chemie.uni-halle.de

*Corresponding author

Table of contents

1 Synthesis 3

1.1 Synthesis of S_4N_4 3

1.2 Synthesis of $(SN)_x$ 3

2 NMR spectroscopic characterisation 4

2.1 NMR spectra of products of the S_4N_4-IL-reaction 4

2.2 NMR spectra of products of ^{15}N labeled S_4N_4-IL-reaction 6

3 ESI-ToF-MS spectrometry 11

4 UV/Vis spectroscopy 12

5 EPR spectroscopy 13

6 Alternative reaction mechanisms 15

1 Synthesis

1.1 Synthesis of S_4N_4

The synthesis of S_4N_4 and $S_4{}^{15}N_4$ was realized with a three-neck flask which was filled with chloroform (400 mL). We added 40 mL S_2Cl_2 to the solvent and equipped the flask with a stirrer, a reflux condenser, and a gas inlet pipe. Furthermore, we flashed the solution with NH_3 (or $^{15}NH_3$) for 10 min and cooled with an ice bath. Unused $^{15}NH_3$ was collected in a rubber ball and re-used for the reaction. We finalized the reaction for the last 4 h with unlabeled NH_3. The reaction solution was purified with distilled water and filtered of NH_4Cl. The filtrate (which was a mixture of sulfur and S_4N_4) was purged with N_2 and dried at room temperature. In the end, the last purification step was realized via sublimation at T = 30 °C and 2 – 3 mbar.

1.2 Synthesis of $(SN)_x$

For the $(SN)_x$ synthesis we used the prepared and cleaned S_4N_4 (^{15}N labelled or unlabeled) from chapter 1.1. The S_4N_4 was sublimated under vacuum at elevated temperatures over silver wool. On the silver wool initially forms Ag_2S, which acts as catalyst for the conversion of S_4N_4 to S_2N_2 in the gas phase. Afterwards, the S_2N_2 crystallized in a cooling trap occupied with liquid nitrogen. The topochemical ring opening polymerization to $(SN)_x$ happens for three days. The crystals are reserved in the dark over several weeks at room temperature to complete the polymerization.

2 NMR spectroscopic characterisation

2.1 NMR spectra of products of the S_4N_4-IL-reaction

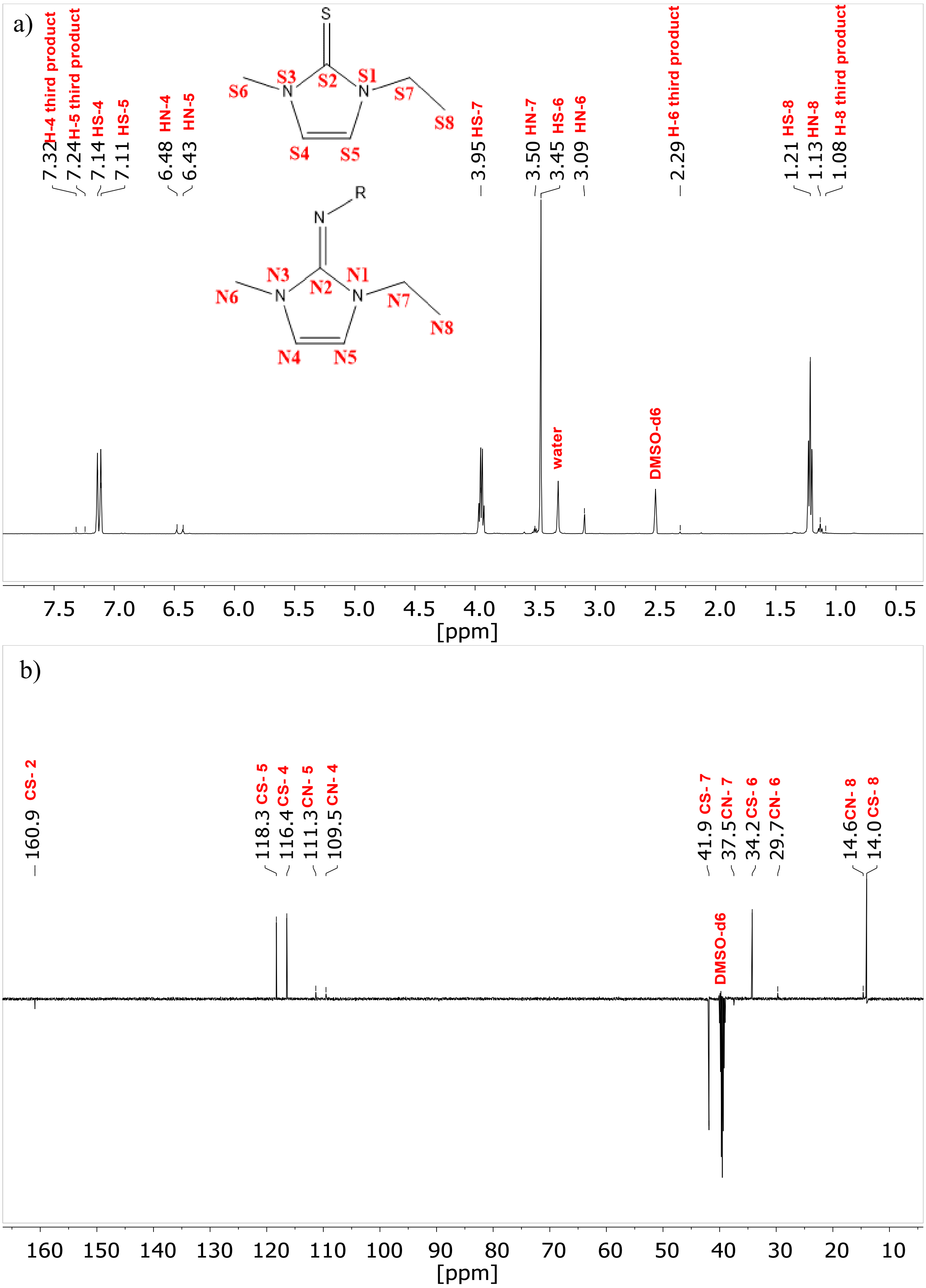

Figure S1: a) ^{1}H NMR and b) ^{13}C NMR spectra of the purified products of the S_4N_4 reaction with [EMIm][OAc].

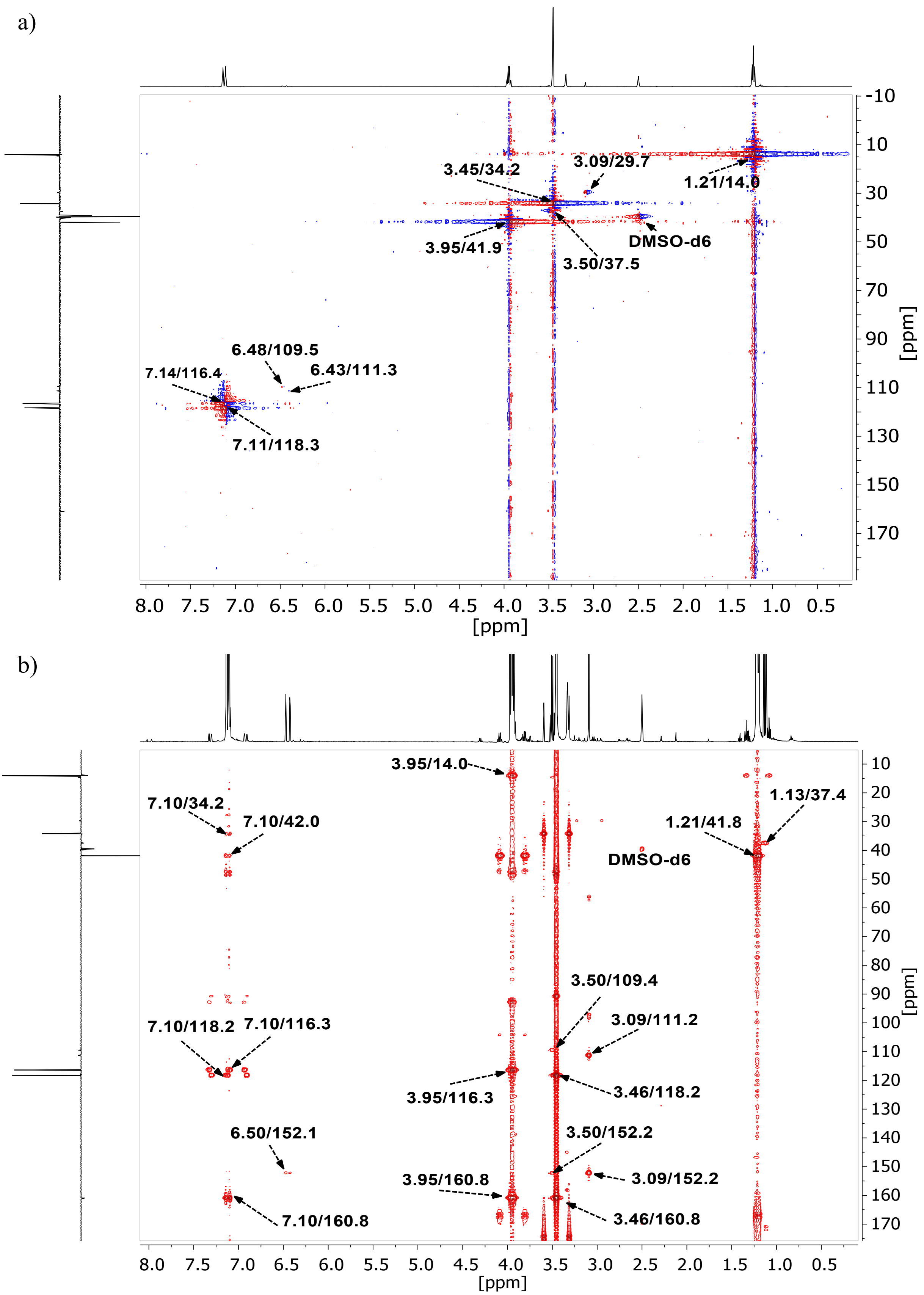

Figure S2: a) ^{1}H-^{13}C HSQC and b) HMBC NMR spectra of products of the S_4N_4 reaction with [EMIm][OAc].

2.2 NMR spectra of products of ^{15}N labeled S_4N_4-IL-reaction

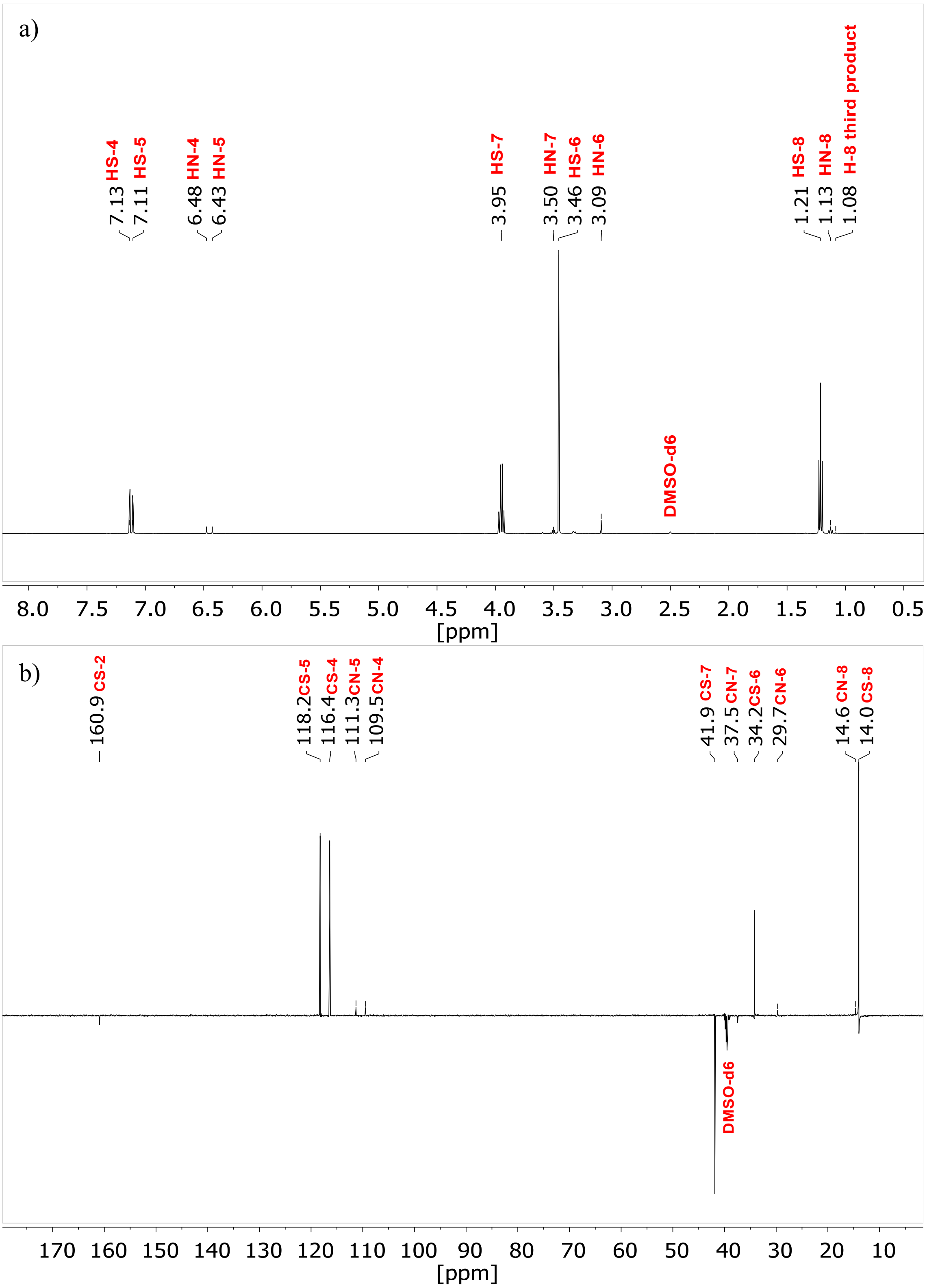

Figure S3: a) ^{1}H and b) ^{13}C NMR spectra of purified products of the $S_4{}^{15}N_4$ reaction with [EMIm][OAc].

a)

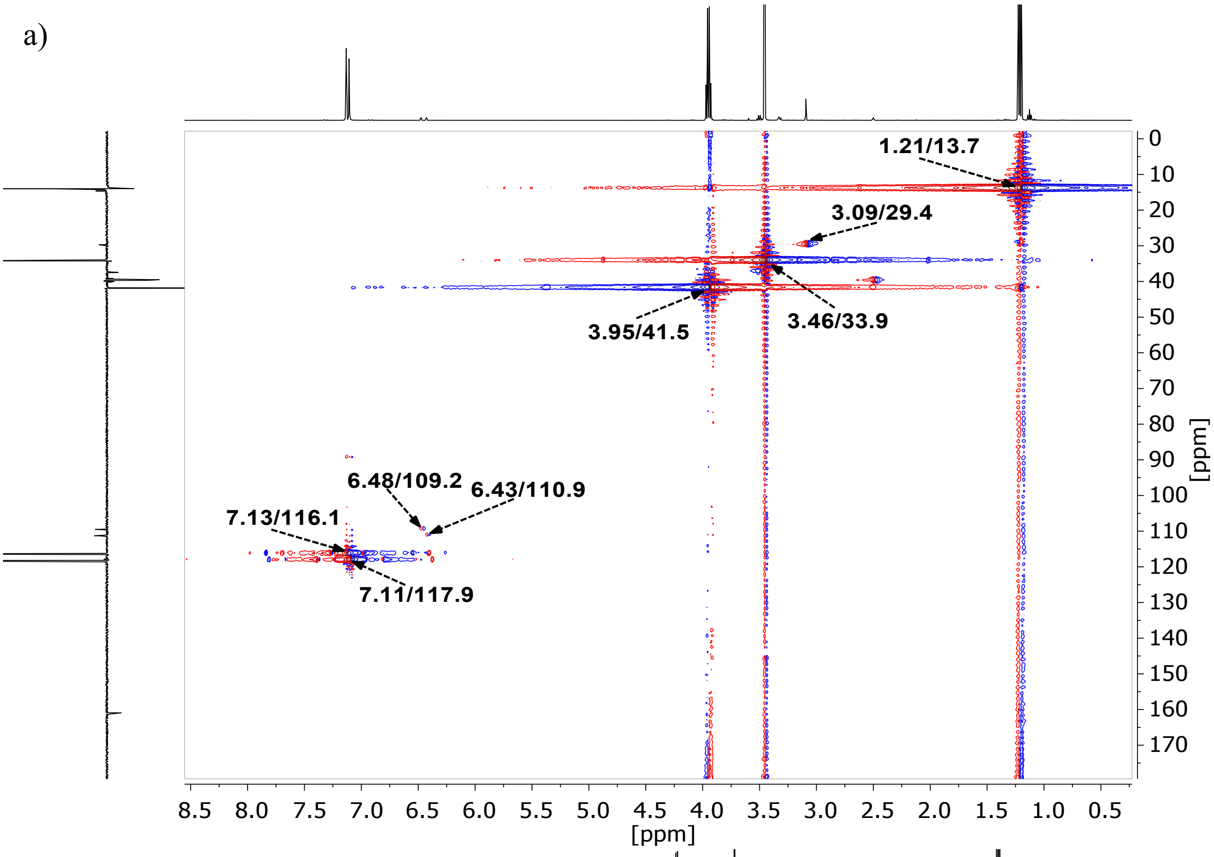

b)

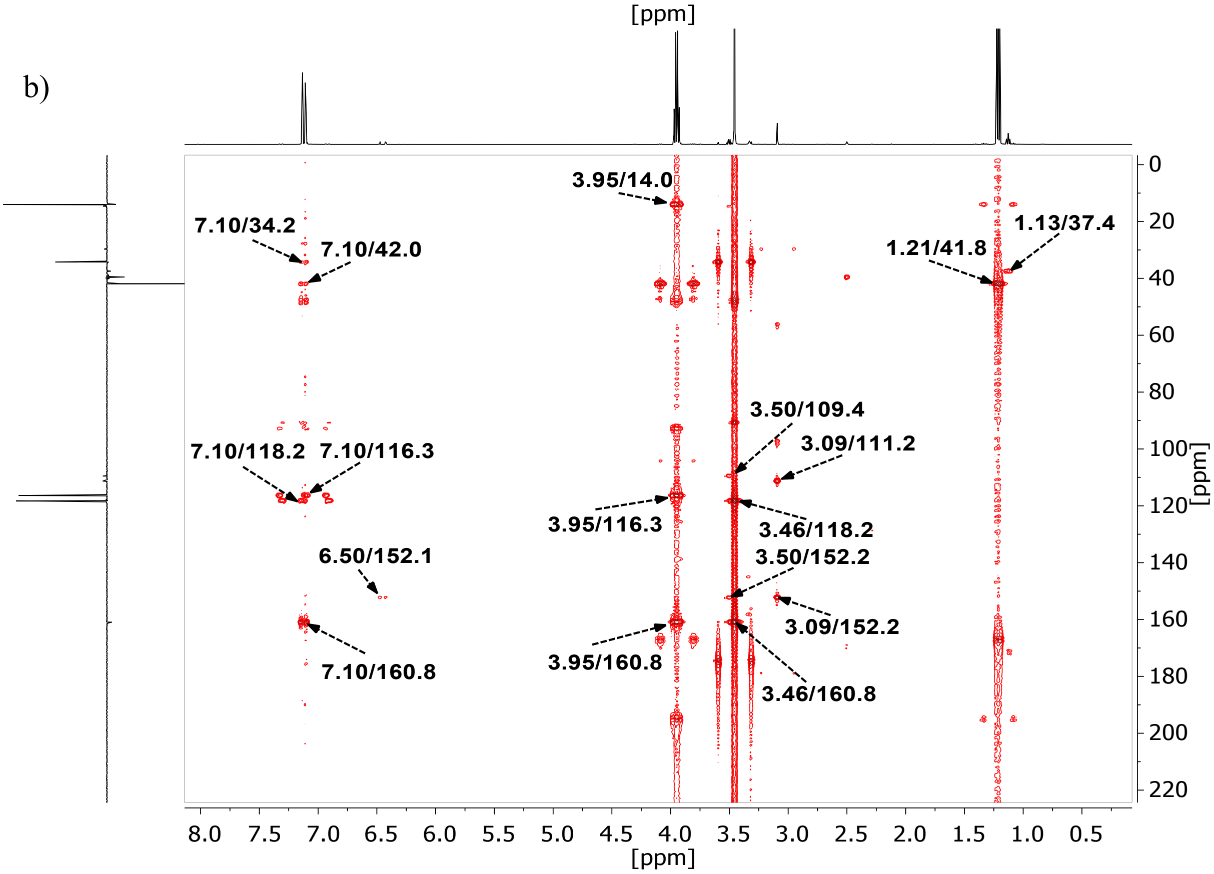

Figure S4: a) ^{1}H-^{13}C HSQC and b) HMBC NMR spectra of products of the $S_4{}^{15}N_4$ reaction with [EMIm][OAc].

Table S1: Chemical shifts of products of the S_4N_4 and $(SN)_x$ reaction with [EMIm][OAc] in the same IL compare with the chemical shifts of the purified products.

**Proton**	**Chemical shift (*multiplet*) [ppm]**			
	$(SN)_x$ + IL	S_4N_4 + IL	Products from $(SN)_x$	Products from S_4N_4
H-2	10.18 (*s*)	10.17 (*s*)	-	-
H-4 + H-5	8.04 (*s*) + 7.87 (*s*)	8.04 (*s*) + 7.88 (*s*)	-	-
HS-4 + HS-5	7.01 (*d*) + 6.97 (*d*)	7.01 (*d*) + 6.98 (*d*)	7.14 (*d*) + 7.11 (*d*)	7.14 (*d*) + 7.11 (*d*)
HN-4 + HN-5	6.19 (*d*) + 6.12 (*d*)	6.19 (*d*) + 6.13 (*d*)	6.48 (*d*) + 6.43 (*d*)	6.48 (*d*) + 6.43 (*d*)
H-6	3.59 (*s*)	3.59 (*s*)	-	-
HS-6	2.94 (*s*)	2.95 (*s*)	3.46 (*s*)	3.45 (*s*)
HN-6	-	-	3.09 (*s*)	3.09 (*s*)
H-7	3.87 (*q*)	3.87 (*q*)	-	-
HS-7	3.39 (*q*)	3.39 (*q*)	3.95 (*q*)	3.95 (*q*)
HN-7	-	-	3.50 (*q*)	3.50 (*q*)
H-8	0.88 (*t*)	0.89 (*t*)	-	-
HS-8	0.59 (*t*)	0.60 (*t*)	1.22 (*t*)	1.21 (*t*)
HN-8	0.50 (*t*)	0.51 (*t*)	1.13 (*t*)	1.13 (*t*)
H-10	1.15 (*s*)	1.15 (*s*)	-	-
H-4 third product	-	-	7.31 (*d*)	7.31 (*d*)
H-5 third product	-	-	7.24 (*d*)	7.24 (*d*)
H-6 third product	-	-	2.30 (*s*)	2.30 (*s*)
H-8 third product	-	-	1.08 (*t*)	1.08 (*t*)

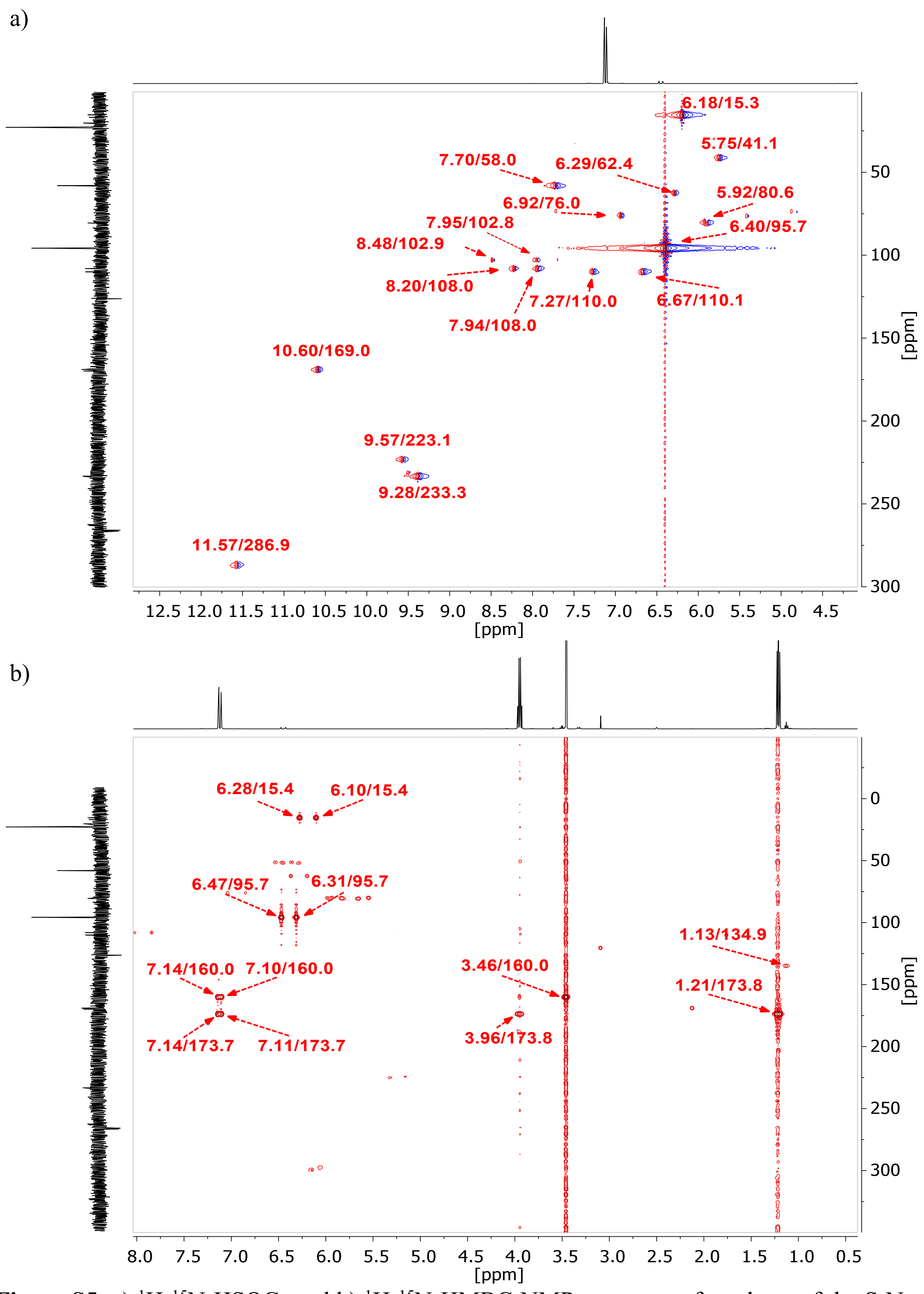

Figure S5: a) ^{1}H-^{15}N-HSQC- and b) ^{1}H-^{15}N-HMBC-NMR spectrum of products of the S_4N_4-[EMIm][OAc] reaction after purification.

Scheme S1: Possible tautomeric forms of EMIm-NSN and other side-products generating ^{15}N NMR signals.

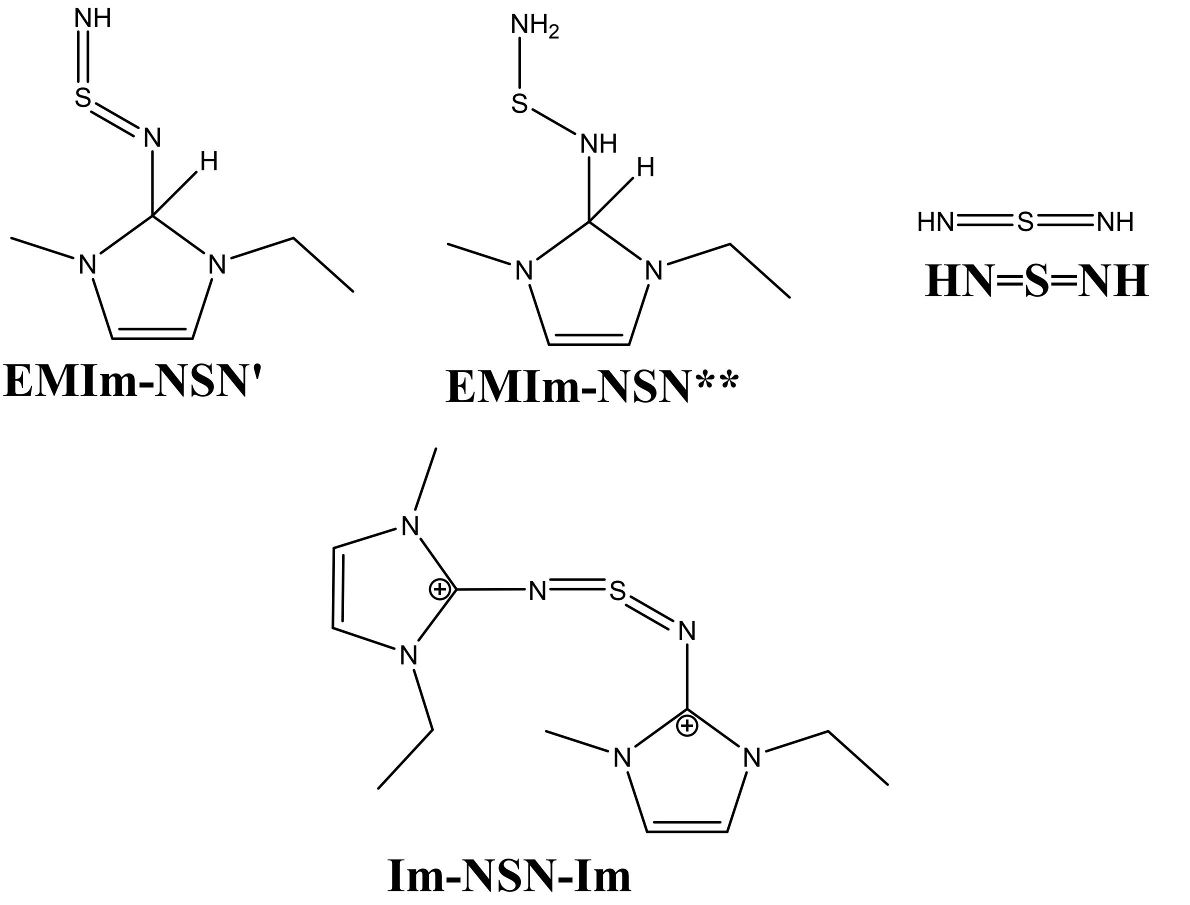

3 ESI-ToF-MS spectrometry

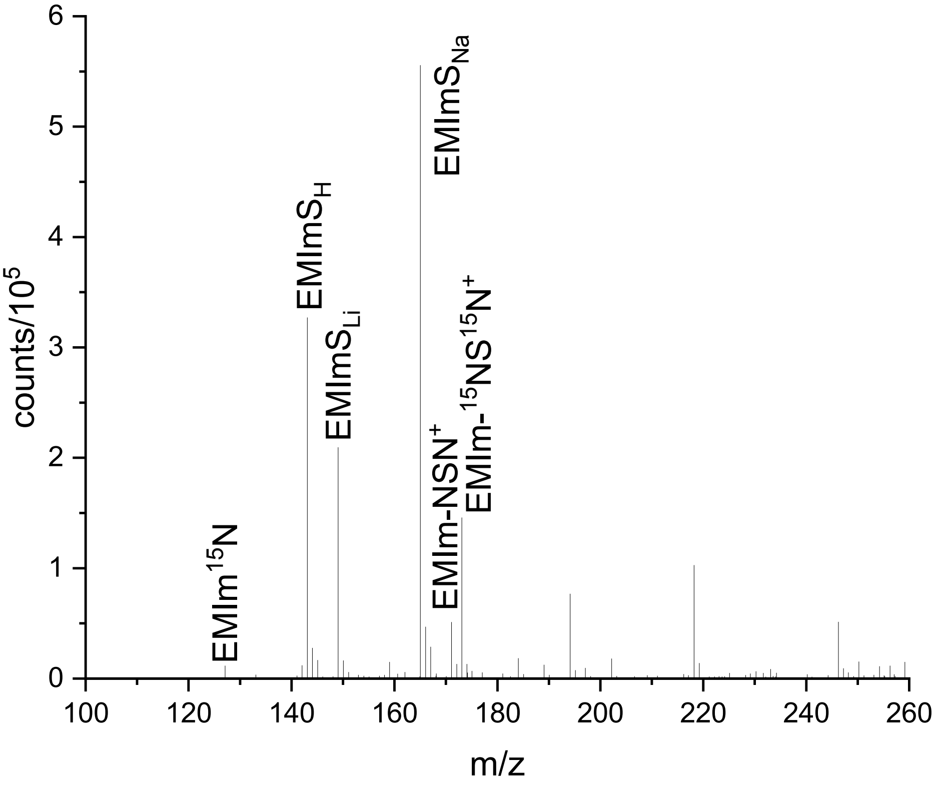

Figure S6: ESI-ToF-MS spectrum of the products of the reaction $(S^{15}N)_x$ with [EMIm][OAc] after the cleaning process.

4 UV/Vis spectroscopy

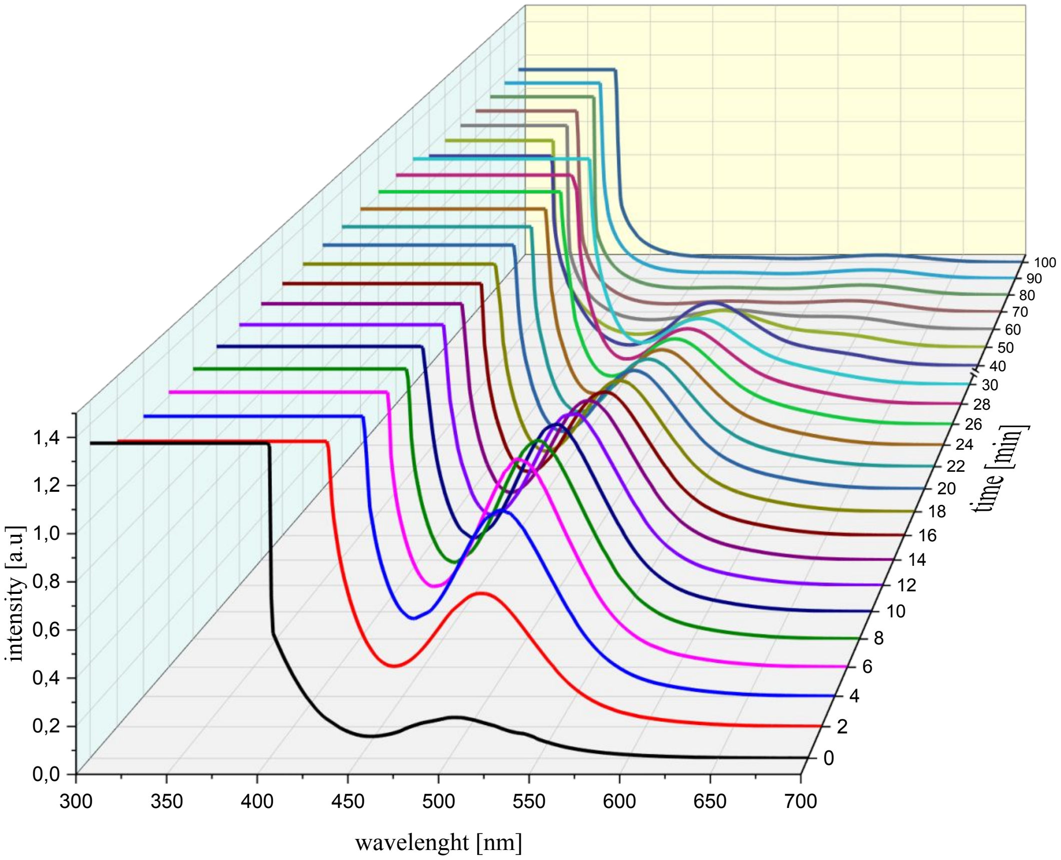

Figure S7: Complete time dependent UV/Vis spectra of S_4N_4 (1 mg) in [EMIm][OAc] (0.1 mL) and DMSO (2 mL).

5 EPR spectroscopy

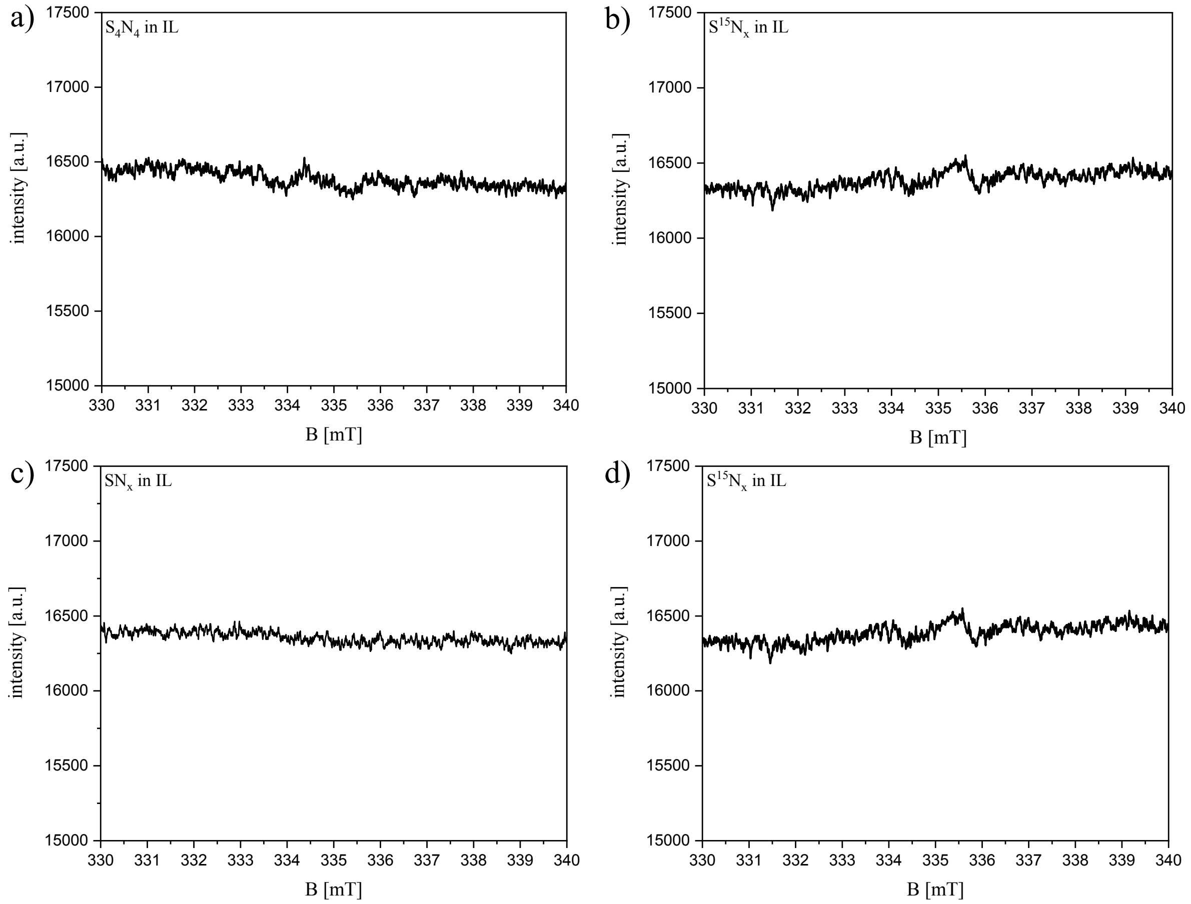

Figure S8: EPR spectra without spin trap of a) S_4N_4 in IL, b) $S_4{}^{15}N_4$ in IL, c) $(SN)_x$ in IL and d) $(S^{15}N)_x$ in IL.

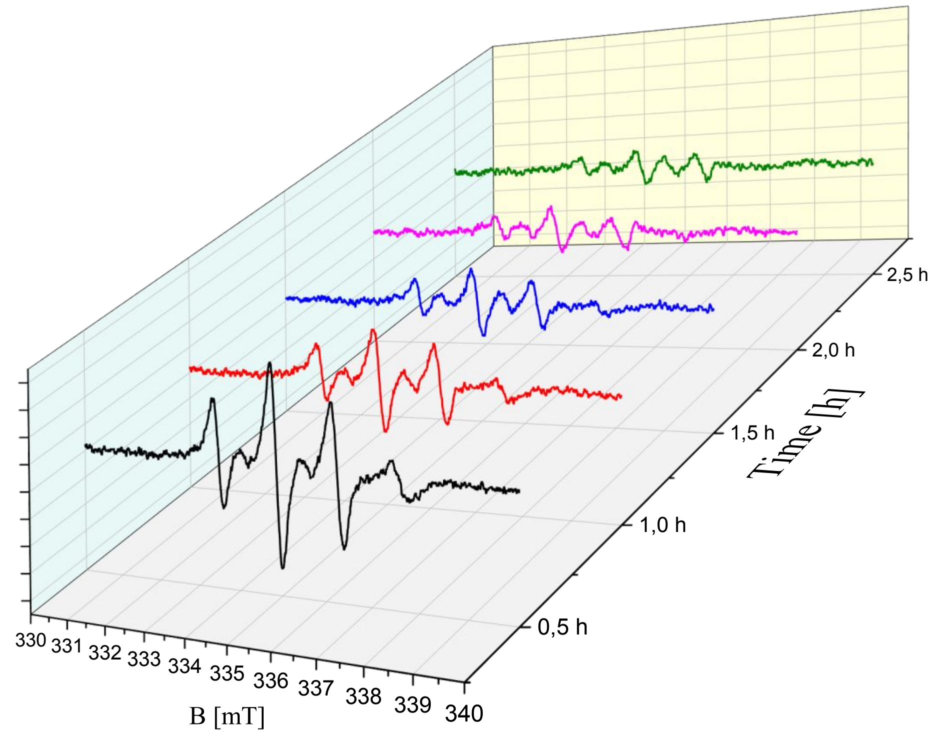

Figure S9: EPR spectra of $(SN)_x$-IL-system with spin trap over the time.

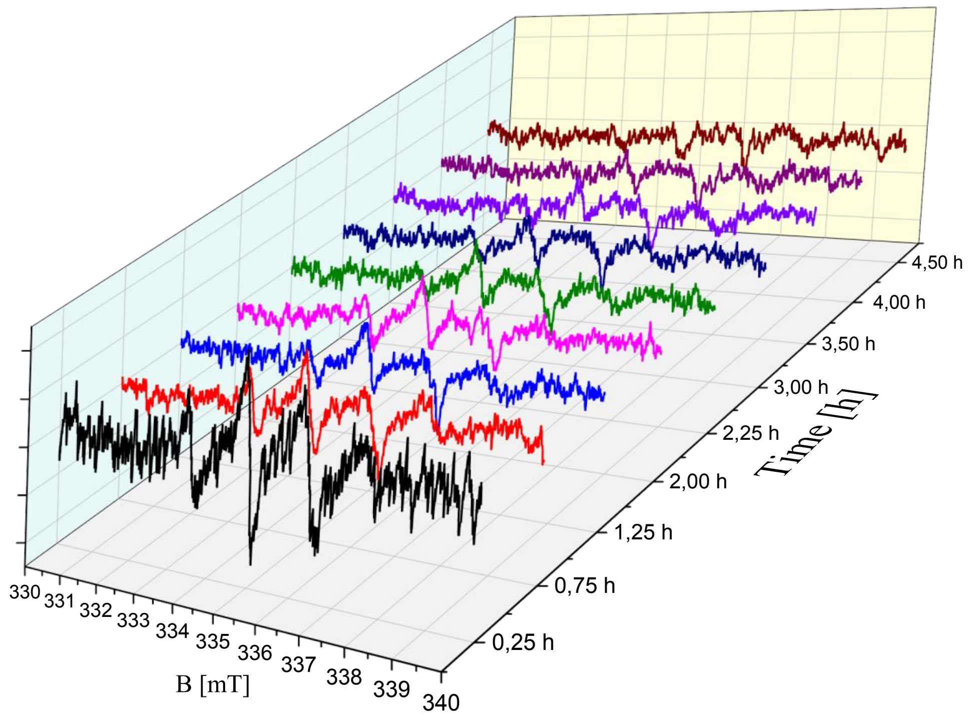

Figure S10: EPR spectra of $(S^{15}N)_x$-IL-system with spin trap over the time.

6 Alternative reaction mechanisms

Reaction step III':

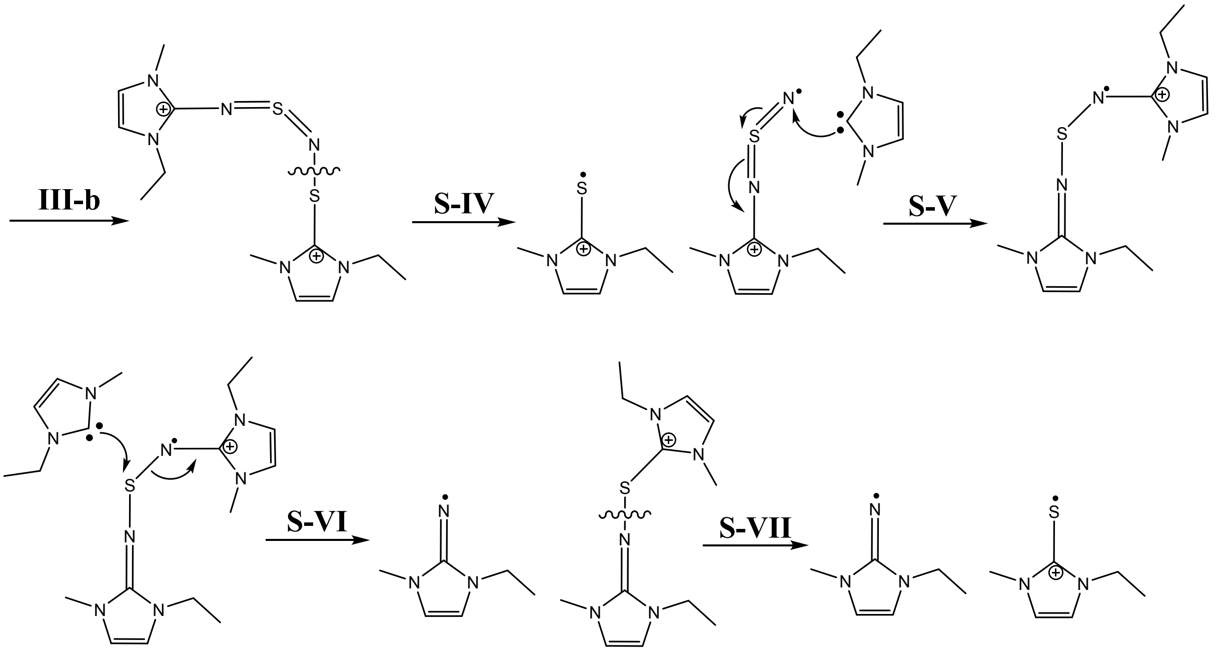

The Im-NSN-S-Im compound of step **III-b** breaking up between sulfur and nitrogen atoms and produce via step **S-IV** two radicals intermediates. In step **S-V** a carbene attacked the nitrogen, produce a diimidazole intermediate which was attacked with another carbene on sulfur position (step **S-VI**). This step result to an imine radical and a diimidazole-NS-intermediate. This broken-up into two radical species like step **S-VII**.
